# Recycling of Polyurethane via Mechanocatalytic Methanolysis/Hydrolysis

**DOI:** 10.1002/cssc.202500253

**Published:** 2025-04-09

**Authors:** Bolun Wang, Joel Britschgi, Nguyen Khang Tran, Ivana Jevtovikj, Piyush Ingale, Cansu Mai, Stephan Andreas Schunk, Ferdi Schüth

**Affiliations:** ^1^ Department of Heterogeneous Catalysis Max‐Planck‐Institut für Kohlenforschung Kaiser‐Wilhelm‐Platz 1 45470 Mülheim an der Ruhr Germany; ^2^ Hte GmbH The High Throughput Experimentation Company Kurpfalzring 104 69123 Heidelberg Germany; ^3^ BASF SE Carl‐Bosch‐Straße 38 67056 Ludwigshafen Germany; ^4^ Institute of Chemical Technology University Leipzig Linnéstraße 3 04103 Leipzig Germany

**Keywords:** base catalysis, chemical recycling, hydrolysis, mechanochemistry, polyurethane

## Abstract

Polyurethane (PU) is the 6th most produced plastic on a global basis, and thus one of the most important targets in the field of recycling of plastic waste. The glycolysis of PU is currently considered the most promising pathway toward industrial implementation. However, the energy consumption during the process, the cost of the excess glycol relative to PU, and the potentially reduced quality of polyol products resulting from glycol residues may still limit the speed of implementation of PU chemical recycling processes from lab to the pilot plant scale. Therefore, an alternative route for PU depolymerization is explored using mechanochemistry and catalysis. In this work, recovery of up to 86% of soluble polyol by mechanocatalytic methanolysis/hydrolysis of NaOH‐impregnated commercial PU product (household sponge), with a Cu/MgAlO_*x*_ co‐catalyst below 100 °C, is described. The recycled polyol can serve as new raw material and has been successfully used as feedstock for the resynthesis of PU. The low reaction temperature, reduced volume of solvent, and easy separation of products could make this novel chemical recycling methodology an attractive alternative to the conventional solvolysis pathways.

## Graphical abstract

In this study, it is shown that up to 86% of soluble polyol can be recovered from the hydrolysis of polyurethane using a ball milling‐assisted process below 100 °C in the presence of NaOH, a small amount of H_2_O and a Cu/MgAlO_*x*_ co‐catalyst.

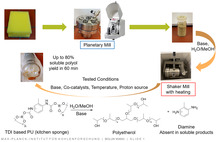

## 1 Introduction

Polyurethane (PU) synthesized from a variety of polyols and isocyanates shows very diverse polymer properties is an advanced material with many applications and as a result is globally the 6th most produced plastic.^[^ ^1^ ^]^ With an annual consumption of 3.81 million tons (Mt) in Europe^[^ ^2^ ^]^ and about 20 Mt worldwide, PU has become one of the most important end‐of‐life plastics that needs to be properly recycled.^[^ ^3^ ^]^ Although an increasing number of post‐consumer plastics were collected across Europe, only 34.6% (10.2 Mt) of the 29.5 Mt collected waste plastics were recycled in 2020 and the rest ended up in incineration (12.4 Mt) and landfill (6.9 Mt).^[^ ^2^ ^]^ Only ≈1% of waste plastics was utilized as a feedstock via chemical recycling,^[^ ^4^ ^]^ as most of the recycled plastic waste was simply mechanically reproduced.^[^ ^5^ ^]^ However, the thermoset PU, which has a cross‐linked structure, cannot easily be reshaped through physical recycling processes.^[^ ^6^ ^]^

An ideal chemical recycling process would directly reproduce monomers, or monomer precursors, required for polymer synthesis, preserving the elements and energy invested in the production loop of plastics for a circular economy. Considering the similarly stable covalent bonds inside and outside their repeat units, it is hardly feasible to selectively recover monomers from vinyl polymers like polyethylene, polypropylene, polystyrene, and polyvinyl chloride. However, unlike the most common polyolefins, PU is a polymer linked by carbamate bonds formed by condensation reactions. Thus, in principle, the higher electrophilicity of the carbamate bonds may enable the selective cleavage of the polar linking bonds to recover monomers.^[^ ^6^, ^7^ ^]^

One already successful case of selective cleavage of heteroatomic linkages is the chemical recycling of polyethylene terephthalate (PET), which has been well‐studied to recover its monomer.^[^ ^8^ ^]^ In contrast, the chemical recycling process of PU is still facing substantial obstacles with respect to the balance between the quality and cost of the products, and thus chemical PU recycling has not passed the pilot plant scale except for an industrialized implementation in H&S Anlagentechnik GmbH from Germany.^[^ ^1^, ^9^ ^]^

The ongoing development of chemical recycling pathways for PU has resulted in approaches such as hydrogenolysis,^[^ ^10^ ^]^ hydrolysis,^[^ ^11^ ^]^ aminolysis,^[^ ^12^ ^]^ phosphorolysis, and glycolysis.^[^ ^1^, ^9^, ^13^ ^]^ In general, after being cleaved through hydrogenolysis at a mild temperature (130–200 °C), the carbamate bond in PU will be broken to yield polyol and amine or urea. Pincer complexes of transition metals can efficiently promote the hydrogenation of carbamate bonds, but the expensive molecular catalysts can hardly be recovered from the products. In analogy to hydrolysis, the cleavage of the carbamate bond can be facilitated by other nucleophiles, using amine, phosphate, or glycol as cleavage reagents. Among these methods, the glycolysis of PU, especially the split‐phase glycolysis process with crude glycerol, is considered the most promising pathway toward industrial implementation from an environmental, technical, and economic point of view.^[^ ^1^, ^9^ ^]^ However, the energy consumption during the process, the cost of the over stoichiometric amounts of glycol required, and the potentially reduced quality of polyol products resulting from the glycol residue still limit the market competitiveness of these chemical recycling processes of PU.

The chemical recycling of PU via mechanochemistry is a promising alternative process to overcome the aforementioned limitations. Although the pulverization of rigid PU by ball milling has been studied for enhancing the quality of physically recycled PU,^[^ ^14^ ^]^ the direct recycling of monomers from PU via ball milling is not reported yet. A twin screw reactive extruder was employed for the partial hydrolysis of thermosetting PU without any catalyst in 1999.^[^ ^15^ ^]^ After extrusion at 200–260 °C, less than 30% of PU was converted into soluble species. The mechanocatalytic depolymerization of lignocellulose has proven to be a powerful methodology to convert the natural polymers into water‐soluble oligosaccharides.^[^ ^16^ ^]^ Without compromising the good mass transfer properties of catalytically active species and polymer, the solvent‐free nature of the mechanical process makes it much easier to separate the soluble products from unconverted plastic waste. The energy consumption of the reaction can also be reduced by the introduction of surface defects and active sites created by the mechanoactivation, which has been validated by the significantly decreased reaction temperature required in the CO oxidation,^[^ ^17^ ^]^ preferential CO oxidation,^[^ ^18^ ^]^ ammonia synthesis,^[^ ^19^ ^]^ coupling reactions,^[^ ^20^ ^]^ and the hydrogenation of various types of solids with carbon backbone to CH_4_^[^ ^21^ ^]^ via ball milling. The mechanochemical depolymerization as an emerging technique^[^ ^22^ ^]^ has been widely applied in the recycling of PET,^[^ ^8^, ^23^ ^]^ polystyrene,^[^ ^24^ ^]^ polyethylene,^[^ ^25^ ^]^ and other plastics at room temperature.^[^ ^26^ ^]^ Beyond the application, thermodynamics and kinetics of mechanochemical reactions have been studied with modeling methods to elucidate the role of mechanical forces in the reactions.^[^ ^27^ ^]^ The successful application of mechanochemical depolymerization at kilogram scale with Simoloyer mills or planetary mills suggests that such recycling processes have the potential to be operated at industrial scale.^[^ ^23^, ^28^ ^]^ Therefore, it appeared promising to approach recovery of monomeric polyol via the mechanocatalytic depolymerization of thermoset PU with lower reaction temperature, cheaper cleavage reagents, and easier separation of products. In this study on the chemical recycling of commercial PU sponge via mechanocatalytic methanolysis and hydrolysis, up to 86 wt% soluble polyols can be recovered by hydrolysis of flexible PU in a shaker mill at 90 °C in 90 min.

## 2 Results and Discussion

**Scheme 1:**
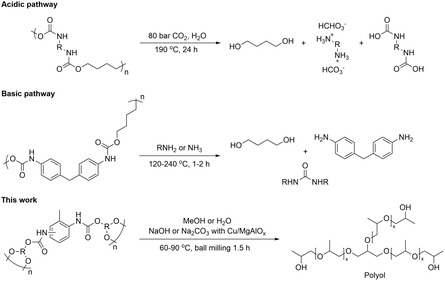
Reaction pathways for the cleavage of carbamate bonds in PU under acidic or basic conditions.

The formation of amine and alcohol products from the cleavage of carbamate bonds in PU requires two protons, which can be provided by H_2_O, NH_3_/amine, or methanol (MeOH)/glycols. The proton transfer to the carbamate bond can be promoted with acid or base catalysts. The hydrolysis pathway under neutral conditions at 200 °C was studied in the early 1970s for the recycling of PU foam in automobiles.^[^ ^29^ ^]^ Even if catalyzed by a strong inorganic base such as NaOH, the hydrolysis of PU normally still requires high reaction temperatures (150–240 °C) and hours of degradation time.^[13a]^ The insolubility of PU in water might be the main reason for such harsh conditions. A very recent work uses organic bases to catalyze the hydrolysis of PU foam dissolved in ionic liquid (IL) below 100 °C, but the cost of IL still limits its industrial application.^[^ ^30^ ^]^ The hydrolysis promoted by carbonic acid also requires high CO_2_ pressure as well as a long reaction time (**Scheme** 1).^[^ ^31^ ^]^ The acidic solution can fix the amine products by the formation of amine salts or carbamic acid, reducing the amine impurities in the recovered polyol.^[^ ^32^ ^]^ Companies like RAMPF Eco Solutions GmbH and H&S Anlagentechnik GmbH have utilized acidolysis to recover aromatic amine‐free (<0.05 wt%) polyol at 200 °C.^[32a,32c]^ However, the low hydroxyl value resulting from acidolysis makes the product only suitable for the synthesis of flexible PU whereas rigid PU requires a higher hydroxyl value that is normally obtained from glycolysis.^[32c]^ In comparison, the aminolysis or ammonolysis of PU under basic conditions can be conducted at relatively milder conditions.^[^ ^12^ ^]^ The additional cost for the consumption of amine/ammonia cleavage reagents and the separation of urea by‐products make such processes less competitive compared to the glycolysis pathway.^[13a]^ Recently, the alcoholysis of polymers connected by heteroatomic linkages with methanol/ethanol has been reported to be a promising pathway for the recycling of monomers via depolymerization under mild conditions (<80 °C).^[^ ^33^ ^]^ Previous work on the methanolysis of thermoplastic PU used temperatures above 200 °C to reach the supercritical state of methanol.^[^ ^34^ ^]^ Using *tert*‐butoxide as a base catalyst, up to 85% diol monomer can be recovered by methanolysis of thermoplastic PU at 65 °C in 20 h.^[^ ^35^ ^]^ Ma et al. recently showed a catalytic process that combines methanolysis and hydrogenation of thermoplastic PU in THF under CO_2_/H_2_ atmosphere at 200 °C, yielding amines and lactones.^[^ ^36^ ^]^ In order to further reduce the cost of PU recycling, ball milling‐assisted methanolysis and hydrolysis of commercial thermoset PU sponge catalyzed by common inorganic bases, that is, NaOH and Na_2_CO_3_ below 90 °C was studied in this work. This could allow the direct recovery of polyetherol, which is the major building block of PU.

### 2.1 Methanolysis/Hydrolysis of PU via Ball Milling

To make the recycling process more applicable to the realistic waste plastics problem, a commercial kitchen sponge, which contains 63 wt% polyol, 25 wt% diaminotoluene moieties, and an undetermined amount of additives, is selected as the thermoset PU substrate. The yellow part of the kitchen sponge was shredded into powder by planetary ball milling (Scheme S1). The conversion of the carbamate bond during the pulverization process is negligible. To ensure the uniform interaction between the PU powder and NaOH during the solid‐phase reaction, PU powder was impregnated with 10 wt% NaOH in aqueous solution. The prepared PU‐NaOH mixed solid is labeled as PUiNaOH‐10 and used as the substrate in a shaker mill, combined with a ceramic heating plate, for the methanolysis and hydrolysis reactions at different temperatures.

**Figure 1:**
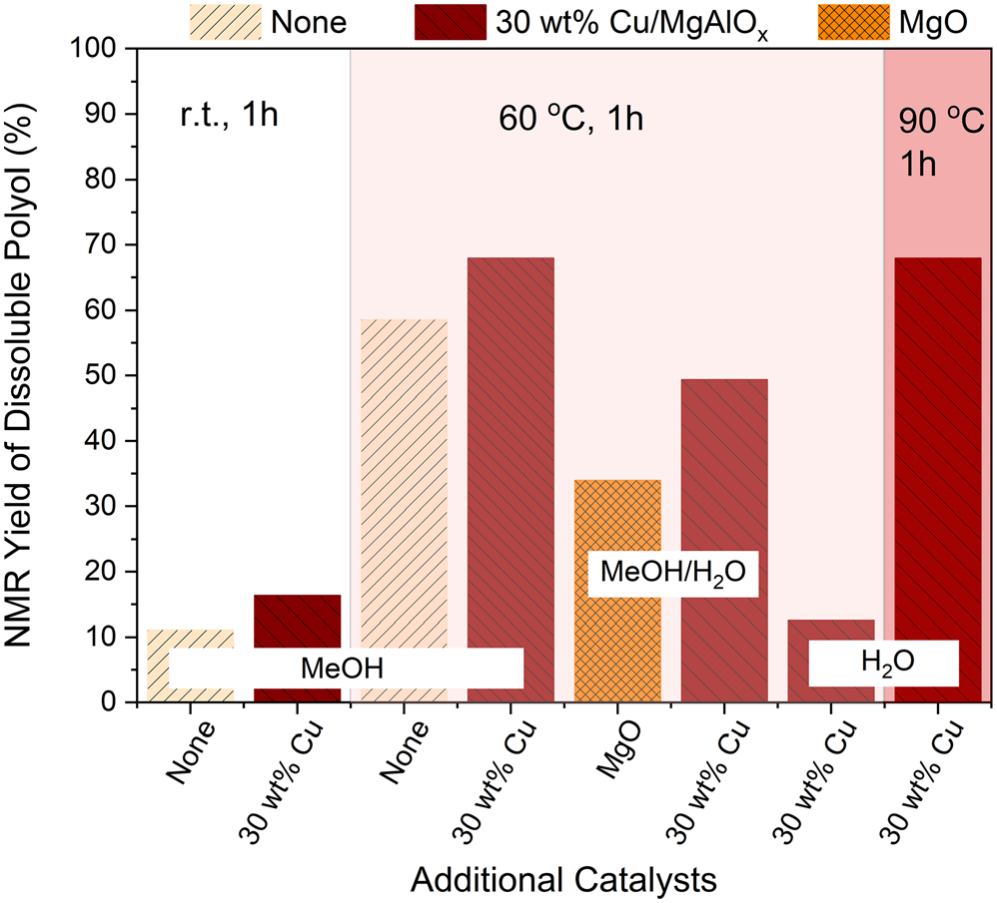
NMR‐based yield of soluble polyols recovered from the methanolysis and hydrolysis of PUiNaOH‐10 in a shaker mill at different temperatures. “None” means there is no additional catalyst used in addition to NaOH.

In general, methanolysis reactions proceed easier than the hydrolysis of PU (**Figure** **1**). Even at room temperature, the presence of only 10 wt% impregnated NaOH is sufficient to promote the cleavage of the carbamate bond and recover 11% of polyol from 0.2 g PU with 200 μL MeOH. When 30 wt% Cu/MgAlO_*x*_ is used as a co‐catalyst, the recovery yield of the polyol is slightly increased to 16%. When the reaction temperature is raised to 60 °C, up to 68% of polyol can be recovered with Cu/MgAlO_*x*_, which is 10% higher than using NaOH alone.

Water is a cheaper and environmentally more friendly proton source compared to MeOH. However, once H_2_O is used, even just using a 100 μL 1:1 (v/v) mixture of H_2_O and MeOH, the yield of polyol obtained using the Cu catalyst significantly decreases to 49%.

Considering that water is more polar than MeOH, it could adsorb on the active sites of the solid catalyst more strongly, thus making the active sites harder to be accessed by the carbamate bonds in PU, a similar solvents effect of polarity is also reported in the hydrogenation of tertiary amides.^[^ ^37^ ^]^ The supported Cu on Mg‐Al oxide (Mg: Al = 70:30), a catalyst also used for the so‐called “Guerbet reaction”, and therefore can be considered as a well‐known solid catalyst for its capability to promote the hydrogen transfer reaction.^[^ ^38^ ^]^ When water is used as the proton source for the hydrolysis of PU, Cu particles are proposed to activate H_2_O, similar to the action of supported Cu catalysts in the water gas shift reaction^[^ ^39^ ^]^ and facilitate proton transfer. The promotion effect of Cu is validated by the control experiment using NaOH with MgO as catalysts, which yields only 34% soluble polyol with a 1:1 (v/v) mixture of H_2_O and MeOH at 60 °C, 15% lower than the yield obtained in the presence of copper. When solely 100 μL H_2_O is used as the proton source, the hydrolysis of PU becomes even more challenging. Only 12% polyol is recovered, using Cu/MgAlO_*x*_ and NaOH at 60 °C. The yield can be improved to 68% by increasing the reaction temperature to 90 °C, possibly because higher temperatures can promote the adsorption/desorption equilibria of H_2_O. This temperature dependency is different from the hydrolysis of PET in a similar shaker mill;^[8c]^ the depolymerization rate did not significantly increase in this case when the reaction temperature was increased from 30 to 90 °C.

**Figure 2:**
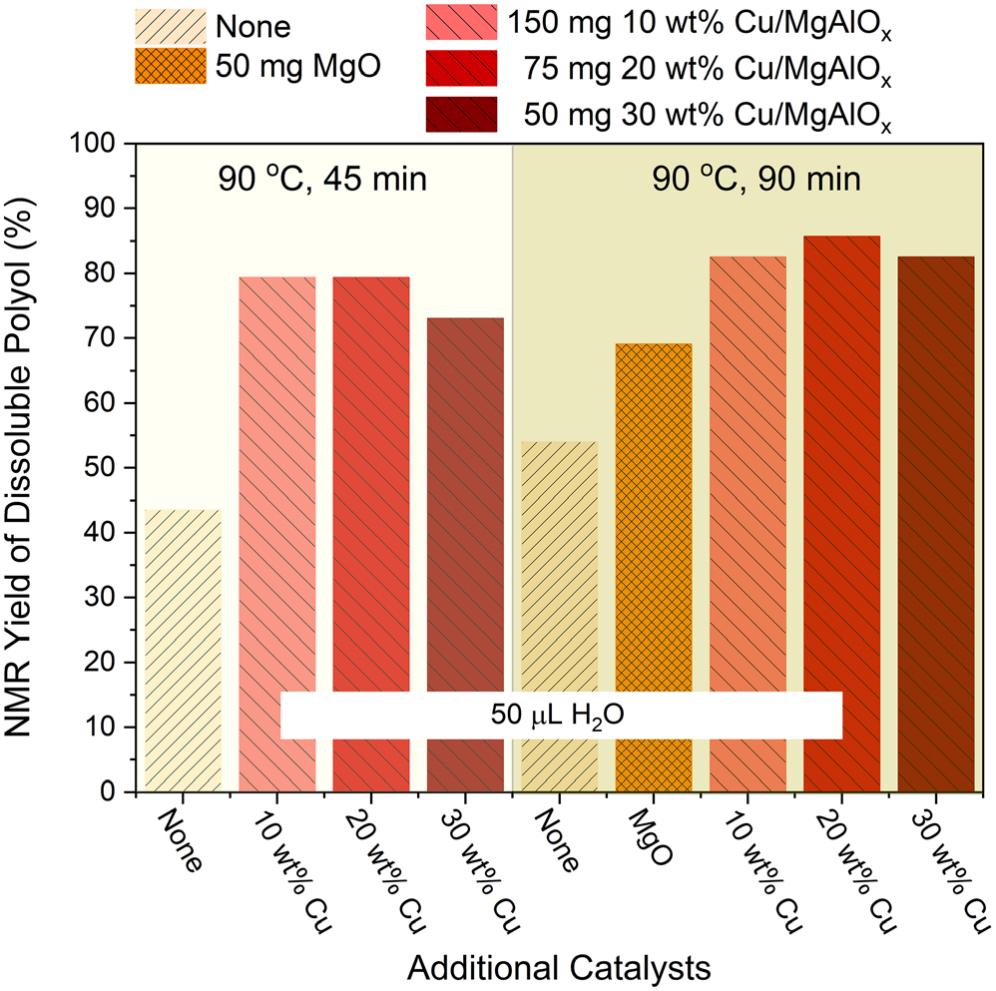
NMR‐based yield of soluble polyols recovered from the hydrolysis of PUiNaOH‐10 in a shaker mill at 90 °C with different loadings of Cu on Mg–Al oxide. “None” means there is no additional catalyst used in addition to NaOH. Different amounts of Cu catalysts were used to keep the amount of Cu constant.

The catalytic performance of Cu/MgAlO_*x*_ is further optimized by controlling the particle size of Cu particles with different loading on the support (**Figure** **2**). A series of Cu/MgAlO_*x*_ are prepared with the same method varying the Cu loading from 10 to 30 wt%. The XRD patterns show that the crystallite size of the Cu metal particles determined from line broadening increases with higher loading (Figure S2, Supporting Information). When 50 μL H_2_O was used as a proton source at 90 °C, these three Cu catalysts showed a similar yield of polyol together with NaOH as catalysts. Up to 79% polyol can be recovered from the hydrolysis of PU with 20 wt% Cu/MgAlO_*x*_ via ball milling in 45 min, which almost doubles the yield compared to using NaOH alone. The recovery yield of polyol can be slightly increased to 86% with 20 wt% Cu/MgAlO_*x*_ when the reaction time is prolonged to 90 min, whereas using NaOH alone and NaOH with MgO show 54% and 69% yield of polyol, respectively. Reducing the NaOH content in the polyurethane powder to 1 wt% (PUiNaOH‐1) lowered the polyol yield from 86% to 44% under mechanochemical hydrolysis at 90 °C for 90 min, which suggests an adequate amount of inorganic base is essential for effective depolymerization. The structure of the recovered polyol is identical to the commercial polyetherol (Lupranol 2074) according to the ^1^H NMR spectra whereas the content of diaminotoluene is too low to be detected in the soluble polyol (Figure S3, Supporting Information). The superior catalytic performance of 20 wt% Cu on Mg–Al oxide is further validated by the hydrolysis of PU at 75 °C (Figure S4, Supporting Information). The yield of polyol obtained with 20 wt% Cu/MgAlO_*x*_ and NaOH is 67%, 10 to 23% more than with the other two Cu catalysts. Moreover, the yield is almost four times higher compared to using NaOH alone. In comparison, less than 7% polyol can be recovered from the hydrolysis of PU catalyzed by NaOH in 10 mL water at room temperature and at 90 °C (Table S1, Supporting Information), validating the advantage of ball milling‐assisted hydrolysis of PU at lower reaction temperature.

### 2.2 Using Na_2_CO_3_ instead of NaOH

**Figure 3:**
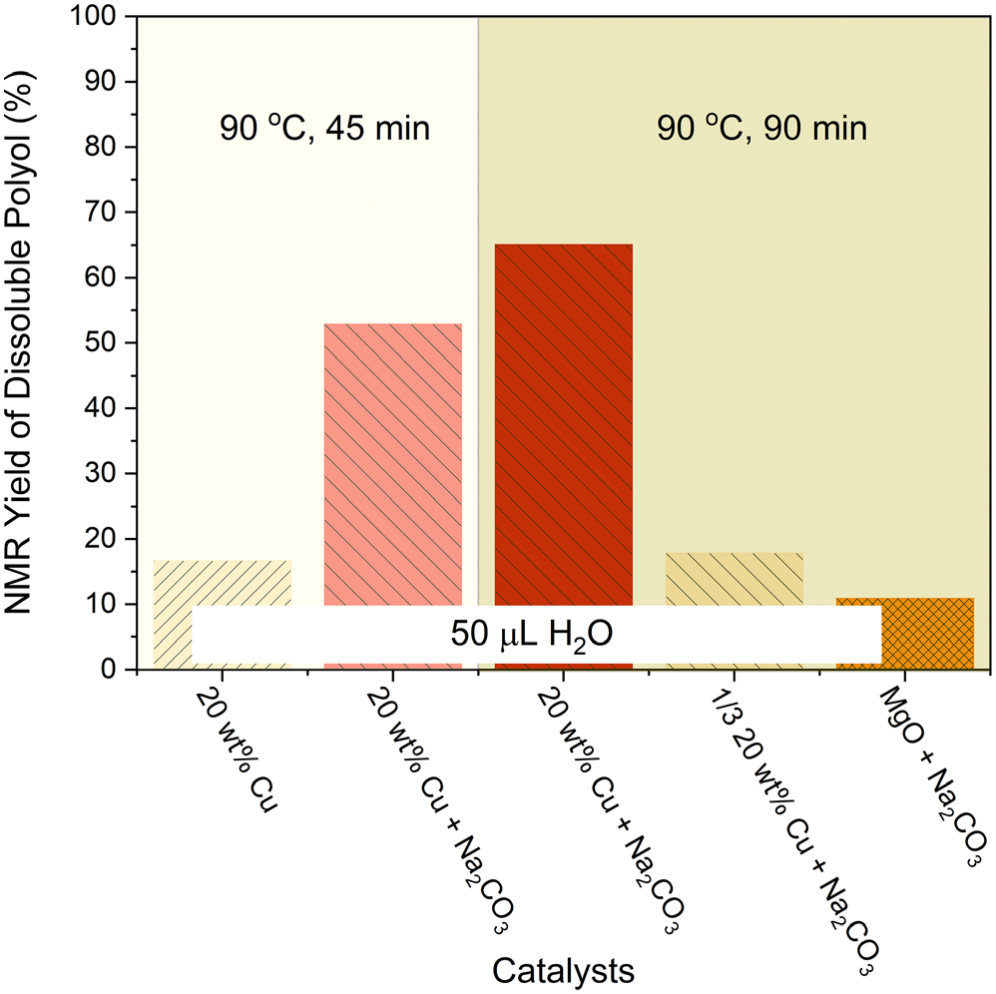
NMR‐based yield of soluble polyols recovered from the hydrolysis of PU powder in a shaker mill at 90 °C without NaOH.

The inorganic base is necessary for this particular hydrolysis process of PU. The yield of polyol decreases from 79% to 17% if 20 wt% Cu/MgAlO_*x*_ is used alone (**Figure** **3**) as the catalyst for the hydrolysis via ball milling at 90 °C for 45 min. Because NaOH is strongly hygroscopic and reacts with CO_2_, the NaOH pellet needs to be uniformly pre‐mixed with PU powder by impregnation and kept under Ar before the hydrolysis reaction. This additional complex impregnation step increases the overall cost of this chemical recycling process. In order to explore how important partial conversion of NaOH to Na_2_CO_3_ by absorbing the CO_2_ in the air would be with respect to depolymerization activity, the effect of using Na_2_CO_3_ as a weaker base to replace NaOH was studied. Na_2_CO_3_ is stable in air and could directly be used in the hydrolysis of PU via ball milling.

When 20 wt% Cu/MgAlO_*x*_ is used together with Na_2_CO_3_ instead of NaOH at 90 °C, the recovery yield of polyol from the hydrolysis of PU is 53% and 65% for 45 and 90 min, respectively (Figure 3). Reducing the 20 wt% Cu/MgAlO_*x*_ loading from 75 mg to 25 mg lowered the polyol yield from 65% to 18% under mechanochemical hydrolysis at 90 °C for 90 min. Only 11% of polyol can be recovered from PU when MgO and Na_2_CO_3_ are used as catalysts without Cu, validating again the important role of Cu as the active metal in the hydrolysis of carbamate bonds.

### 2.3 Quality of the Recycled Polyol

Before using the recycled polyols for the resynthesis of PU, their quality is compared with commercial polyetherol (Lupranol 2074) by gel permeation chromatography (GPC), electrospray ionization mass spectrometry (ESI‐MS) and attenuated total reflection‐infrared spectroscopy (ATR‐IR). Three recycled polyol samples obtained from the methanolysis of PUiNaOH‐10 with 30 wt% Cu/MgAlO_*x*_ at 60 °C for 60 min, the hydrolysis of PUiNaOH‐10 with 20 wt% Cu/MgAlO_*x*_ at 90 °C for 45 min and the hydrolysis of PU with Na_2_CO_3_ and 20 wt% Cu/MgAlO_*x*_ at 90 °C for 90 min are labeled as PO‐1, PO‐2, and PO‐3, respectively in the following analysis.

Firstly, the molecular weight distribution was analyzed by GPC with refractive index (RI) and UV detector (Figure S5–S8, Table S2 and S3, Supporting Information). Four major components in the soluble polyol can be identified by GPC with an RI detector. Based on their average molecular weight and the molecular weight distribution of Lupranol 2074, they are assigned to high‐weight polyol (8700–9000 Da), medium‐weight polyol (5000–5300 Da), low‐weight polyol (3000–3300 Da), and polyol fragments resulting from the cleavage of a C—O bond of the polyol (900–1000 Da), respectively. The majority (>75%) of the polyol is recovered with medium weight for all three samples, which shows the similar molecular weight distribution compared to Lupranol 2074 (Figure S8, Supporting Information).^[10d]^ ≈10% of high‐weight polyol is identified in PO‐1 and PO‐3, whereas the high‐weight polyol is absent in PO‐2. PO‐2 and PO‐3 obtained by hydrolysis show less than 5% low‐weight polyol, while PO‐1 resulting from methanolysis shows more (≈10%). All the details of the distribution of polyol fragments detected by the RI detector and UV detector are shown in Tables S2, S3, Supporting Information.

It needs to be pointed out that ≈10% of polyol fragments are also identified in PO‐2 and PO‐3. As its molecular weight is only 1/3 of the low‐weight polyol, the fragment should come from the cleavage of the C—O bond inside the polyol molecule instead of the cleavage of the carbamate bond. The significantly increased content of this fragment determined by the UV detector (Figure S5–S7, Supporting Information) suggests a higher content of the diaminotoluene moiety—responsible for the UV absorption—in the fragment compared to medium‐weight polyol obtained by hydrolysis (PO‐2 and PO‐3). However, the medium‐weight polyol obtained by methanolysis (PO‐1) also shows a strong UV response, indicating that this polyol moiety might still be linked to the diaminotoluene end groups by carbamate bonds to some extent. As the polyol itself is hardly detected by the UV detector, the quantification result of polyol fragments by the RI detector (Table S2, Supporting Information) is more reliable than the UV detector (Table S3, Supporting Information).

PO‐3 is further analyzed with ESI‐MS to validate its structural unit (Figure S9, Supporting Information). Like the commercial polyetherol (Lupranol 2074), the recycled polyol shows a C_3_H_6_O_1_ repeating unit (*m/z* = 58.04), which indicates that the polyol used for the production of commercial PU sponge is synthesized by the polymerization of propene oxide. Diaminotoluene (*m/z* = 123.09) is also identified in the recovered polyol mixture, validating the cleavage of the carbamate bond. In the *m/z* range from 500 to 3000, the molecular weight distribution of recycled polyol slightly moves toward the lower mass range compared to Lupranol 2074. This suggests the cleavage of C—O bonds inside the polyol, which is in line with the GPC results. As ESI‐MS has poor sensitivity in the higher mass range, the medium‐weight and high‐weight polyols that are detected by GPC are not identified by ESI‐MS.

**Figure 4:**
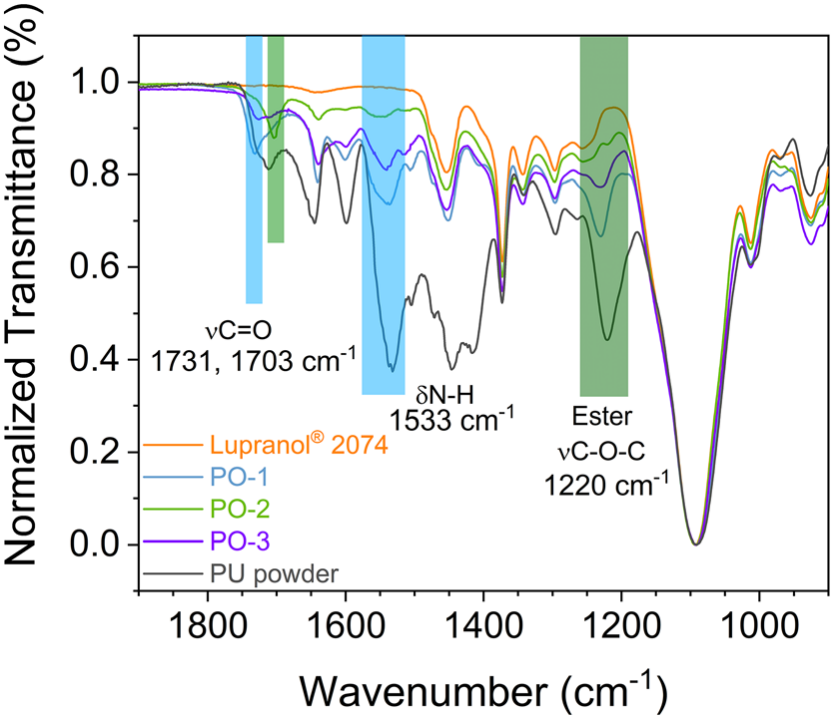
ATR‐IR spectra (900–1900 cm^−1^) of the commercial polyetherol standard (Lupranol 2074), the initial PU powder, and the polyols recycled from the PU powder.

ATR‐IR was employed to analyze the functional groups (**Figure** **4** and Figure S10, Supporting Information), especially the end groups and amine residue in the recycled polyol, which is crucial for the condensation with isocyanate in re‐polymerization of the recovered moieties. The broad absorbance peak (1730–1700 cm^−1^) of the pristine PU can be deconvoluted into two peaks. The peak at 1731 cm^−1^ can be attributed to the ν(C=O) in the ester structure of the carbamate bond, whereas the other peak at 1703 cm^−1^ can be attributed to the ν(C=O) in the urethane bond.^[^ ^40^ ^]^ As expected, the ν(C=O) absorbance at 1731 cm^−1^ and the ester asymmetric ν(C—O—C) absorbance^[^ ^41^ ^]^ at 1220 cm^−1^, which are absent in Lupranol 2074, have almost disappeared in PO‐2 and PO‐3. But PO‐2 also shows significant absorbance of ν(C=O) at 1703 cm^−1^. Although this feature in the carbonyl range was significantly reduced for PO‐1, the polyol recovered via methanolysis at a lower temperature still shows considerable residual C=O species in the ester structure as reflected by the absorbance at 1731 and 1220 cm^−1^.

In general, the content of amine groups in the recycled polyols was strongly reduced compared to the parent PU powder, indicated by the decreased absorbance of the ν(N—H) at 3300 cm^−1^ and the δ(N—H) at 1533 cm^−1^.^[^ ^40^ ^]^ However, the amine groups are not completely removed in the case of PO‐1 and PO‐3. In comparison, PO‐2 shows less residual amine groups and ester moieties, as indicated by the much weaker absorbance at 3300, 1533, and 1220 cm^−1^. The residual diaminotoluene moieties in PO‐1 and PO‐3 are also indicated by the absorbance of ν(C=C) in the aromatic ring at 1645 and 1599 cm^−1^.^[^ ^40^ ^]^ This feature is pronounced in the parent PU powder due to the diaminotoluene moieties, which is negligible in PO‐2. In conclusion, the content of residual amine groups and ester structure in these three polyol samples follows the sequence PO‐1 > PO‐3 > PO‐2. To quantitatively compare the content of residual amine species in the recovered polyols, their nitrogen content was determined by elemental analysis (Table S4, Supporting Information). The quantification results of amine species in terms of nitrogen content are consistent with the results obtained from IR spectra. After depolymerization, the 5.7 wt% nitrogen in the PU powder was reduced to 0.8–2.7 wt% in the recovered polyols, whereas the commercial polyol (Lupranol® 2074) contains 0.4 wt% nitrogen. Assuming the 0.8 wt% nitrogen impurity in PO‐2 exists solely in the form of diaminotoluene, the diamine content is ≈3.5 wt%, which is much lower than the diamine impurity (124.0 mg KOH g^−1^, 13.5 wt%) obtained from the glycolysis of PU at 180 °C for 5 h.^[^ ^42^ ^]^ The deamination of the polyol from glycolysis has been well studied.^[^ ^32^, ^42^ ^]^ Alkene oxides have been employed as deamination agents to diminish the content of diamine impurity below 40 mg KOH g^−1^ (4.3 wt%),^[^ ^42^ ^]^ which is still higher than the amine content in PO‐2.

Compared to the parent PU powder, the number of OH groups is significantly increased in all three recycled polyol samples, indicated by the wide absorbance of ν(O—H) around 3480 cm^−1^. Among the samples, PO‐3 shows the highest content of hydroxyl groups and the lowest concentration of residual carbonyl groups. On the one hand, the amine end groups are not active for the condensation reaction; thus, the residual amine moieties will reduce the quality of re‐synthesized PU. On the other hand, a higher content of hydroxyl groups will make the recycled polyol more active for the condensation with isocyanate. In order to use the recycled polyol as the feedstock for the synthesis of PU, its quality needs to be balanced between the residual amount of the amine moieties/carbonyl groups and the generated amount of hydroxyl groups. Therefore, Na_2_CO_3_ is selected as the base for the hydrolysis of PU to obtain enough polyol of sufficient quality for the re‐polymerization, although it shows an inferior recovery yield of polyol compared to NaOH.

**Figure 5:**
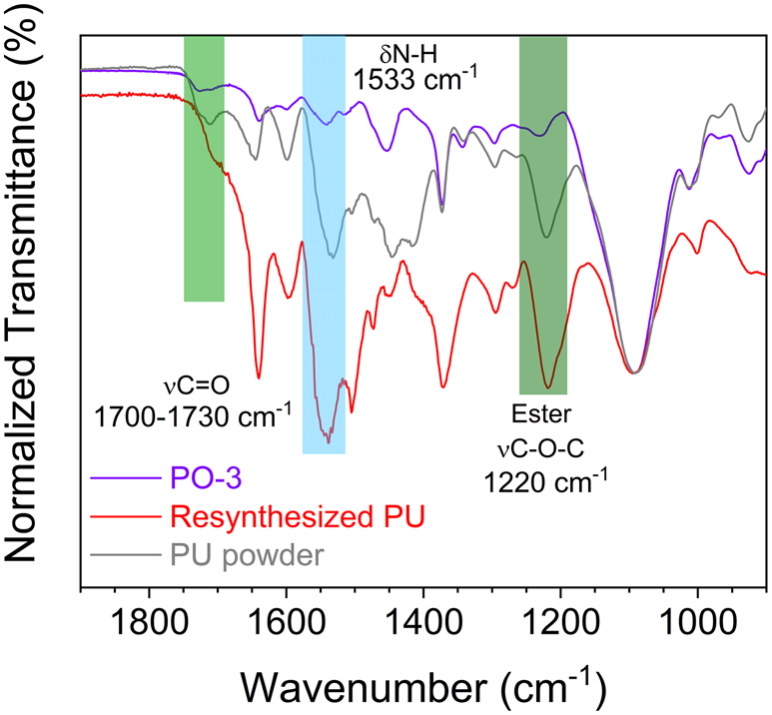
ATR‐IR spectra (900–1900 cm^−1^) of the recycled polyols, the insoluble PU resynthesized from the recycled polyol and TDI, and the initial PU powder.

The hydrolysis of PU with Na_2_CO_3_ and 20 wt% Cu/MgAlO_*x*_ was then scaled up to 0.6 g via ball milling at 90 °C for 90 min to produce enough polyol for the resynthesis of PU and spent catalyst for the catalyst reuse test. Solid PU can be produced from the condensation polymerization of PO‐3 with toluene‐2,4‐diisocyanate (TDI) in DMF at room temperature. As indicated by ATR‐IR, the resynthesized PU shows increased intensity of ν(N—H), ν(C=O), δ(N—H), and ν(C—O—C) at 3300, 1730, 1533, and 1220 cm^−1^, respectively (**Figure** **5** and Figure S11, Supporting Information). Although the synthetic conditions are yet to be optimized for achieving a quality close to the virgin TDI‐PU, the resynthesized PU proves that the recycled polyol can serve as the feedstock for PU production.

### 2.4 Reuse of the Catalysts

**Figure 6:**
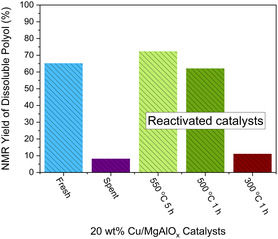
NMR‐based yield of soluble polyols recovered from the hydrolysis of PU powder in a shaker mill at 90 °C with fresh, spent, and 20 wt% Cu/MgAlO_*x*_ regenerated at different temperatures. Na_2_CO_3_ was only added when the fresh Cu/MgAlO_*x*_ was used.

The spent 20 wt% Cu/MgAlO_*x*_ catalyst is recovered together with the base from the scaled‐up reaction. The direct reuse of the mixture of spent catalysts for the hydrolysis of PU without additional Na_2_CO_3_ only produces 8% polyol (**Figure** **6**). However, the deactivated catalysts can be simply regenerated by calcination in air at 550 °C for 5 h. Then the yield of polyol obtained with the regenerated catalysts without additional Na_2_CO_3_ is increased to 72%. As indicated by the XPS spectra (Figure S12–S14, Supporting Information), the deactivation is mainly due to the surface coverage by carbon and amine species (399.8 eV),^[^ ^43^ ^]^ which can be removed by calcination. It also explains why the amine products are not identified in the soluble products, as the amine is adsorbed strongly on the catalysts and cannot easily be extracted by organic solvents. In order to reduce the energy cost of catalyst regeneration, the regeneration of spent catalyst was also carried out at 500 °C and 300 °C for 1 h, respectively. The catalyst reactivated at 500 °C shows a 62% polyol yield, which is comparable to the fresh 20 wt% Cu/MgAlO_*x*_ and Na_2_CO_3_. However, only 11% polyol can be recovered, if the catalyst was reactivated at 300 °C for 1 h, suggesting the incompletely removed PU residue is poisoning the surface of Cu/MgAlO_*x*_.

According to the XRD pattern, the diffraction pattern of Cu metal particles in the fresh 20 wt% Cu/MgAlO_*x*_ has disappeared essentially completely after the reaction (Figure S15, Supporting Information). The emerging diffraction feature of CuO suggests that Cu particles are oxidized during the reaction. After the calcination, the feature of CuO is retained, but with a considerable fraction of metallic Cu. A possible explanation is that the metallic Cu may be located in the core of the Cu‐containing particles and could be protected from oxidation by the oxidized surface layer during the calcination in the air.

## 3 Conclusion

Up to 86% of soluble polyol can be recovered by mechanocatalytic methanolysis/hydrolysis of NaOH‐impregnated commercial PU sponge below 100 °C. The sluggish hydrolysis rate is improved by elevated temperatures and the presence of Cu/MgAlO_*x*_ co‐catalysts. Most of the polyol is recovered with the molecular weight distribution similar to Lupranol 2074, peaking around 5300 Da. The recycled polyol has been successfully used as feedstock for the resynthesis of PU with TDI. While part of the diaminotoluene moiety is still connected to the polyols, especially in the minority (ca. 10%) fragments of the polyols, a trace amount of free diaminotoluene can be detected by ESI‐MS. However, the amount is too small to be separated from the polyol products. Due to the carbonaceous species, including also amines, on the surface, the hydrolysis catalysts are deactivated after the reaction, but can be reused after the calcination in the air without supplying fresh base. The lower reaction temperature, reduced volume of solvent, and easier separation of products could make this novel chemical recycling methodology advantageous compared to the conventional solvolysis pathways.

## Conflict of Interest

The authors declare no conflict of interest.

## Supporting information

Supplementary Material

## Data Availability

The data that support the findings of this study are available from the corresponding author upon reasonable request.
