# Supplementary material for: Recycling of Polyurethane via Mechanocatalytic Methanolysis/Hydrolysis
Source: ChemSusChem. 2025 Apr 9;18(12):e202500253. doi: 10.1002/cssc.202500253 (PMC12175035; doi:10.1002/cssc.202500253)
Supplement: Supplementary file 1 — Supplementary Material [file CSSC-18-e202500253-s001.zip › cssc202500253-sup-0001-SuppData-S1.pdf]

Recycling of Polyurethane via Mechanocatalytic Methanolysis/Hydrolysis

Bolun Wang,[a] Joel Britschgi,[a] Nguyen Khang Tran,[a] Ivana Jevtovikj,[b] Piyush Ingale,[b] Cansu Mai,[b] Stephan Andreas Schunk,*[b, c, d] and Ferdi Schüth*[a]

[a] Dr. B. Wang, Dr. J. Britschgi, N. K. Tran, Prof. Dr. F. Schüth
Department of Heterogeneous Catalysis,
Max-Planck-Institut für Kohlenforschung
Kaiser-Wilhelm-Platz 1, 45470 Mülheim an der Ruhr, (Germany)
Email: schueth@kofo.mpg.de

[b] Dr. I. Jevtovikj, Dr. P. Ingale, Dr. C. Mai, Dr. S. A. Schunk
Hte GmbH, the High Throughput Experimentation Company
Kurpfalzring 104, 69123 Heidelberg, (Germany)

[c] Prof. Dr. S. A. Schunk
BASF SE
Carl-Bosch-Straße 38, 67056 Ludwigshafen, (Germany)

[d] Prof. Dr. S. A. Schunk
Institute of Chemical Technology,
University Leipzig
Linnéstraße 3, 04103 Leipzig, (Germany)

Supporting information for this article is given via a link at the end of the document.

The preparation of PU powder from commercial PU sponge

1.4 g of the commercial PU sponge (Spontex, the yellow soft part) was cut into ca. 8 cm^3 cubes, then transferred into a home-built stainless steel (SS) milling jar (Scheme S1) for the planetary ball milling in a Fritsch Pulverisette 6. Seven 10 mm and two 12 mm SS milling balls (in total ca. 40 g) were placed at the bottom of the milling jar. The milling chamber was then covered with a Teflon-lid with SS filter which only allows the penetration of gases, then the SS jar was sealed by its own lid with six tightened screws by a torque wrench (20 Nm). After being properly sealed, the air inside the milling chamber was replaced to Ar at ambient pressure by connecting the gas valve (Fig. 1a) to a Schlenk line. The milling jar contains PU sponge and milling balls was then mounted to the Fritsch Pulverisette 6 for planetary mill. At room temperature, each batch took 60 minutes milling at 600 rpm, followed by a pause of 10 min to allow cooling, then repeating the 60 minutes milling in a reverse direction. After 6 hours of milling, the PU sponge is completely crushed into fine PU powder for the following study of depolymerization. Shorter milling time may lead to a small amount of incomplete crushed bulk solid, but the milling time has not been optimized, yet. The PU powders are prepared under Ar to avoid the influence from O_2 and moisture during the milling; this may be not necessary for potential industrial application.

The preparation of Cu/MgAlO$_x$ catalysts

The supported Cu catalysts with different Cu loadings (*10-30* wt% Cu/MgAlO$_x$) were prepared using incipient wetness impregnation. A certain amount of $Cu(NO_3)_2$•$3H_2O$ was dissolved in DI water and added dropwise to 25 g of Pural MG 70 and thoroughly mixed. The impregnated material was later dried at 80 °C for 3h and calcined at 350 °C for 4h with 1K/min in muffle furnace under clean dry air. Reduction was done at 400 °C for 12 hours with temperature ramp of 5 K/min.

The impregnation of NaOH to PU powder to prepare PUiNaOH-10

In a 50 mL flask, 1 g of PU powder was mixed with 10 g 1 wt% NaOH aqueous solution. The mixture was stirred with a magnetic bar in the closed flask at room temperature for 2 hours. The water in the flask was then removed by rotation evaporation at 50 °C in 2 hours. The impregnated PU powder (10 wt% NaOH loading, labeled as PUiNaOH-10) was dried in a vacuum oven at 50 °C overnight, then kept under Ar to avoid the absorption of water and CO_2 from air.

The depolymerization of PU powder by shaker mill with ceramic heating box

220 mg of PUiNaOH-10 powder was added into a home-built SS milling jar (Scheme S1), the cleavage reagents were then added according to the desired amount of proton source (H_2O, methanol or their mixture). In order to keep the Cu weight identical in each experiment, 50, 75 and 150 mg of 30, 20 and 10 wt% Cu/MgAlO$_x$ was used, respectively. In case of using Na_2CO_3 instead of NaOH as base, 200 mg PU powder with 40 mg Na_2CO_3 was added to the milling jar with the same following procedure. Three 10 mm SS balls (in total ca. 12 g) were placed at the bottom of the milling jar. After the liquid was added to the mixture dropwise, the powder got

wet but did not form a slurry. The milling chamber was then sealed by its SS lid with a polyether ether ketone (PEEK) flat O-ring. The milling jar was then mounted to a modified Retsch MM400 shaker mill with home-built ceramic heating box covering the milling jar. The heating box is connected to a temperature controlling unit which can adjust the box temperature according to the target temperature set for the thermocouple attached to the milling jar. The shaker mill was started at 25 Hz frequency together with the temperature ramping of the heating box. In order to heat the milling jar up to 90 °C in short time, the temperature for the heating box was set as 240 °C. It usually took less than 15 minutes to reach 60 ^{o}C and roughly 30 minutes to reach 90 °C after the milling jar being heated in the box. When the milling is finished, the milling jar was taken off and opened immediately. The solid products was transferred to a 50 mL centrifuge tube with 30 mL ethyl acetate (EA) before the milling jar was cooling down. The centrifuge tube was placed in an ultrasonic bath at room temperature for 5 min to completely extract the soluble products. The dissoluble products were then separated from the solid residue by a centrifuge at 9000 rpm for 10 minutes, leading to a clear yellow solution. The solvent was then removed by rotation evaporation at 45 °C in 30 minutes to obtain dissoluble polyol (P_L). The residual solid (P_S) from the reaction was dried together with P_L in a vacuum oven at 50 °C overnight.

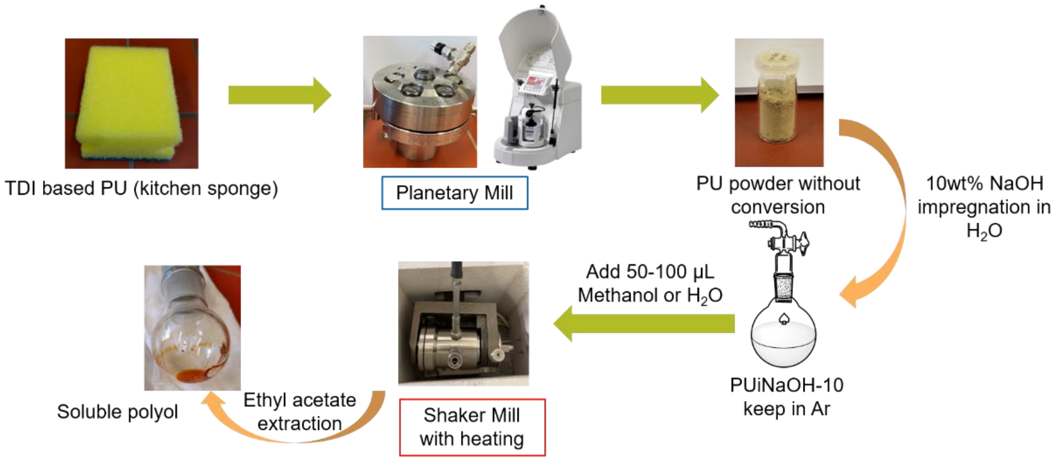

Scheme S1. The workflow of the methanolysis/hydrolysis of PU with ball milling processes to recover soluble polyol from commercial kitchen sponge.

Characterization methods

Elemental analysis was performed by Mikroanalytisches Laboratorium Kolbe (Fraunhofer Institut UMSICHT, Osterfelder str. 3, D-46047 Oberhausen).

NMR spectra were obtained on a Bruker AV300nano spectrometer. All spectra were collected at 25 °C in standard 5 mm tubes containing $CDCl_3$ volumes of about 0.5 mL.

The electrospray-ionization mass spectra (ESI-MS) were collected on a Q Exactive Plus (Thermo Scientific, Bremen, Germany), using an electrospray ionization source in positive mode [ESI(+)] at 4 kV ionization voltage and in negative mode [ESI(-)] at 3,5 kV ionization voltage. Mass spectra were recorded over a range of 100 < m/z <6000 with a resolution setting of R = 280000 (full width at half-maximum at m/z = 200).

The functional groups of the samples were analyzed by Fourier transform infrared (FTIR) spectroscopy using a Perkin Elmer-Spectrum Two (Perkin Elmer, Waltham, USA) spectrometer equipped with an attenuated total reflectance (ATR) accessory. Samples were directly measured on the surface of the ATR crystal (diamond) at room temperature, recording 16 scans per spectrum with a resolution of 4 cm^{-1} in the range of 400-4000 cm^{-1}.

Crystal structures of Cu/$MgAlO_x$ catalysts were analyzed by powder X-ray diffraction (XRD) using Stoe theta/theta diffractometer with the Bragg−Brentano geometry using Cu Kα1/2 radiation (1.541862 Å).

X-ray photoelectron spectroscopy (XPS) was performed with a custom spectrometer from SPECS GmbH equipped with a Phoibos 150 1D-DLD hemispherical energy analyzer. The monochromatized Al Kα X-ray source (E = 1486.6 eV) was operated at 15 kV and 200 W. For high-resolution scans, the pass energy was set to 20 eV and for survey scans to 50 eV. The medium area mode was used as lens mode. The base pressure in the analysis chamber was 5×10^{-10} mbar during the experiment. Spectra were referred to C 1s at 284.5 eV to account for charging effects.

Gel permeation chromatography (GPC) analysis was employed to determine the molecular weight distribution (MWD) of recovered Polyols. The recovered polyol samples were dissolved in tetrahydrofuran (THF) at a concentration of 10 mg/mL and then filtered (pore size 0.45 µm). Measurements were performed with an Shimadzu HPLC system (LC-20 series) equipped with a four-columns set (PSS SDV combination mediumlow) from Agilent (1 x SDV Pre-column 3 µm 8x50 mm, 2 x SDV column 3 µm 1000Å 8x300 mm and 1 x SDV column 3 µm 10000Å 8x300 mm), a refractive index detector (RI) and a UV detector (PDA) using THF as eluent at 35 °C (flow: 1 mL/min)Polystyrene standards (from Macherey-Nagel, Germany) with molecular weight ranging from 92 to 15.000.000 were used for MWD calibration. The MW calculation was performed with the Shimadzu GPC post-run analysis.

The quantification of dissoluble polyol with NMR internal standard

According to the elemental analysis of dry PU powder pulverized from sponge, which contains 56.79% C, 8.21% H, 17.87% O and 5.66% N, the pristine PU is comprised of 63 wt% polyol and 25 wt% diaminotoluene moieties, respectively. Therefore, maximum 126 mg polyol can be recovered from 200 mg PU powder that was used as the substrate for depolymerization. According to the literature,[1] the molecular weight of commercial polyetherol (Lupranol® 2074) is around 3210 g/mol. The weight of dry P_L and P_S were measured before further quantification of P_L. 40 mg 1,3,5-Tribromobenzene (Fw: 315) was added to the flask containing P_L as an internal standard for the ^{1}H NMR spectra. The mixture of standard and P_L was dissolved in 2 mL $CDCl_3$, then ^{1}H NMR spectra were measured using a Bruker AV300nano spectrometer. The ratio ***a*** between the features of the nine hydrogen atoms from the three methyl groups in the repeat unit of polyol (Peak 1, blue shade) and the three hydrogen atoms on the benzene ring of standard (Peak 2, red shade) can be calculated from their integration areas (Figure S1). Then the recovery yield (***x***%) of the dissoluble polyol (Fw: 3228, n = 17) to the 126 mg polyol moiety in the 200 mg parent PU can be calculated from the following equation.

$$\boldsymbol{a} = \frac{\boldsymbol{x} \div 100 \times 200 \times 0.63 \times 9 \times 17}{3228} \div \frac{40 \times 3}{315} \quad \textbf{Equation 1.}$$

$$\boldsymbol{x} = \frac{\boldsymbol{a} \times 20 \times 3}{315} \div \frac{9 \times 17 \times 0.63}{3228} \quad \textbf{Equation 2.}$$

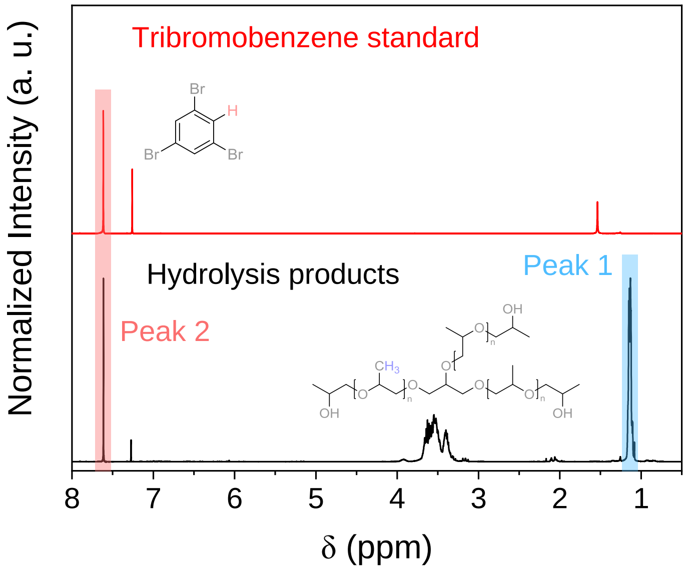

Figure S1. ^{1}H NMR spectra of 1,3,5-Tribromobenzene standard (top, red curve) and the dissoluble polyol recovered from the hydrolysis of PUiNaOH-10 (bottom, black curve). The feature of the nine hydrogen atoms from the three methyl groups in the repeat unit of polyol (Peak 1) are marked in a blue shade and the feature of the three hydrogen atoms on the benzene ring of the standard (Peak 2) are marked in a red shade.

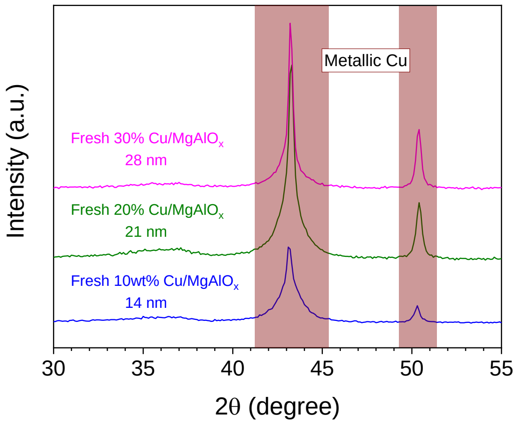

Figure S2. XRD patterns of fresh Cu supported on Mg-Al oxide (Mg:Al = 7:3) catalysts with different loading. The crystallite sizes were estimated based on the peak at 43.2° using the Scherrer equation.

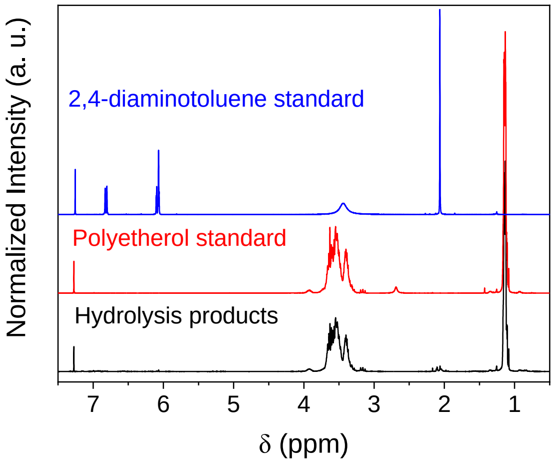

Figure S3. The ^{1}H NMR spectra of 2,4-diaminotoluene, polyetherol (Lupranol® 2074) and the hydrolysis products of PUiNaOH-10 co-catalyzed by Cu/$MgAlO_x$ in the shaker mill at 90 °C for 90 min.

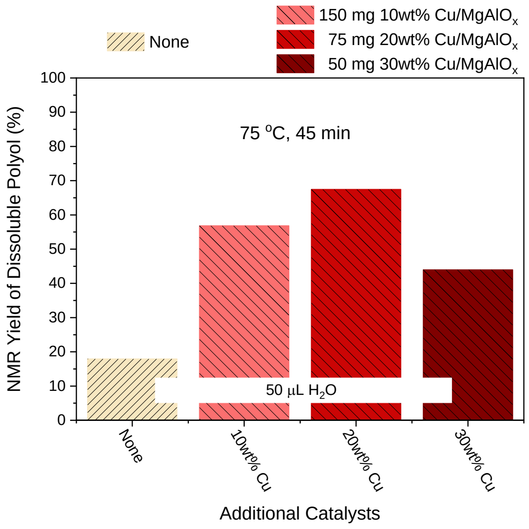

Figure S4. Hydrolysis of PUiNaOH-10 in a shaker mill at 75 °C with different loadings of Cu on Mg-Al oxide. “None” means there is no additional catalyst used in addition to NaOH.

Table S1. The hydrolysis of PU conducted in solution and shaker mill.

Reactor	Conditions	Temperature	Time	Yield[a]
Stirring in a pressure vessel	1 g PU, 10 mL 1 wt% NaOH *aq*	r.t.	2 h	6.3%
	0.2 g PU, 75 mg 20wt% Cu/$MgAlO_x$, 10 mL 1 wt% NaOH *aq*	90 °C	1.5 h	2.9%
Ball milling in a shaker mill	0.22 g PuiNaOH-10, 75 mg 20 wt% Cu/$MgAlO_x$, 50 µL H_2O	90 °C	1.5 h	86%

[a] Yield of soluble polyol calculated from the ^1H NMR spectra with tribromobenzene as internal standard.

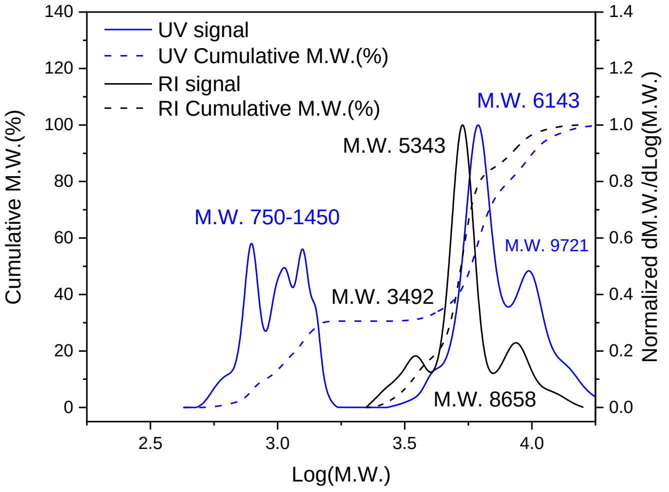

Figure S5. GPC determined molecular distribution of the soluble polyol recovered from the methanolysis of PUiNaOH-10 and 30 wt% Cu/$MgAlO_x$ via ball milling at 60 ºC for 60 min (PO-1). The curve in blue is recorded by UV detector and the curve in black is recorded by RI detector.

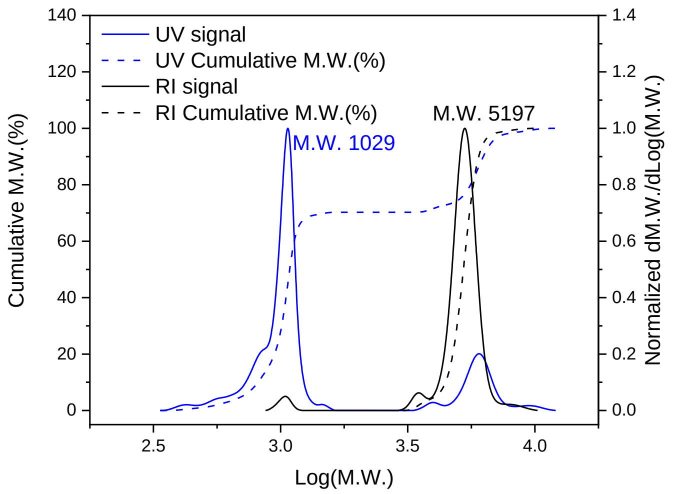

Figure S6. GPC determined molecular distribution of the soluble polyol recovered from the hydrolysis of PUiNaOH-10 and 20 wt% Cu/MgAlO$_x$ via ball milling at 90 ºC for 45 min (PO-2). The curve in blue is recorded by UV detector and the curve in black is recorded by RI detector.

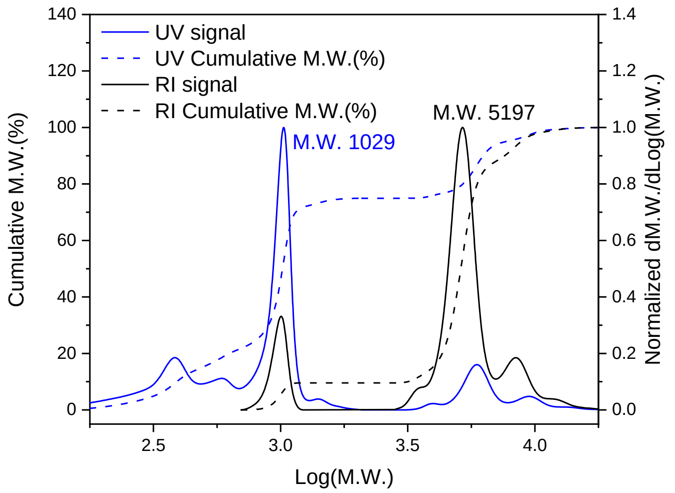

Figure S7. GPC determined molecular distribution of the soluble polyol recovered from the hydrolysis of PU with Na_2CO_3 and 20 wt% Cu/MgAlO$_x$ via ball milling at 90 ˚C for 90 min (PO-3). The curve in blue is recorded by UV detector and the curve in black is recorded by RI detector.

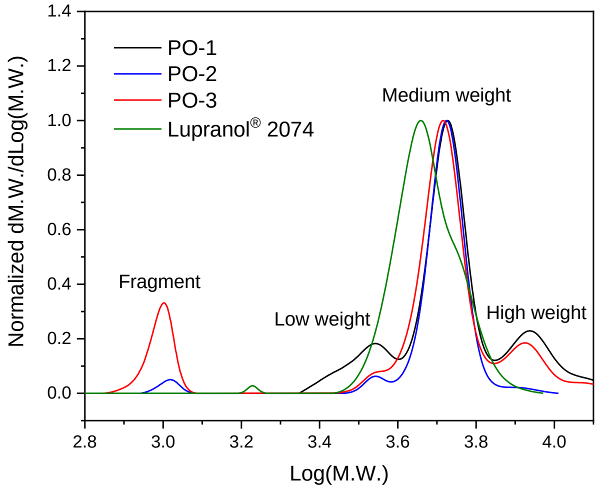

Figure S8. Molecular distribution of polyol fragments determined by GPC with RI detector. The blue dashed curve shows the soluble polyol recovered from the methanolysis of PUiNaOH-10 and 30 wt% Cu/$MgAlO_x$ via ball milling at 60 ºC for 60 min (PO-1). The black curve shows the soluble polyol recovered from the hydrolysis of PUiNaOH-10 and 20 wt% Cu/$MgAlO_x$ via ball milling at 90 ºC for 45 min (PO-2). The red curve shows the soluble polyol recovered from the hydrolysis of PU with Na_2CO_3 and 20 wt% Cu/$MgAlO_x$ via ball milling at 90 °C for 90 min (PO-3). The green curve shows the polyol standard (**Lupranol® 2074**) provided by BASF.

Table S2. Average molecular weight data for each soluble polyol fractions determined by GPC with RI detector.

Polyol Recovery Conditions	Polyol Species Area Ratio (%)	Number Average Molecular Weight (Mn /Da)	Weight Average Molecular Weight (Mw /Da)	Polydispersity Index (PDI)
**PO-1** 68% Polyol recovered from PUiNaOH-10+30 wt% Cu/$MgAlO_x$, 200 µL MeOH, 60 ºC, 60 min	15.3%	8928	9169	1.03
	67.4%	5246	5314	1.01
	17.2%	3222	3278	1.02
**PO-2** 79% Polyol recovered from PUiNaOH-10+20 wt% Cu/$MgAlO_x$, 50 µL H_2O, 90 ºC, 45 min	1.4%	8087	8138	1.01
	92.6%	5215	5273	1.01
	4.2%	3474	3485	1.00
	1.7%	1033	1037	1.00
**PO-3** 65% Polyol recovered from PU+Na_2CO_3+20 wt% Cu/$MgAlO_x$, 50 µL H_2O, 90 ºC, 90 min	12.0%	8771	9043	1.03
	75.5%	5060	5141	1.02
	2.9%	3352	3361	1.00
	9.6%	978	984	1.01
**Lupranol® 2074**	95.9%	4596	4747	1.03
	0.4%	1692	1694	1.00
	3.6%	335	337	1.00

Table S3. Average molecular weight data for each soluble polyol fractions determined by GPC with UV detector (PDA).

Polyol Recovery Conditions	Polyol Species Area Ratio (%)	Number Average Molecular Weight (Mn /Da)	Weight Average Molecular Weight (Mw /Da)	Polydispersity Index (PDI)
**PO-1**<br>68% Polyol recovered from PUiNaOH-10+30 wt% Cu/MgAlO$_x$,<br>200 µL MeOH, 60 ºC, 60 min	20.1%	10310	10678	1.04
	49.3%	5768	5963	1.03
	3.4%	1449	1452	1.00
	8.3%	1253	1256	1.00
	9.0%	1023	1028	1.00
**PO-2**<br>79% Polyol recovered from PUiNaOH-10+20 wt% Cu/MgAlO$_x$,<br>50 µL H_2O, 90 ºC, 45 min	9.9%	749	759	1.01
	55.1%	1060	1068	1.01
	15.1%	705	734	1.04
**PO-3**<br>65% Polyol recovered from PU+Na_2CO_3+20 wt% Cu/MgAlO$_x$,<br>50 µL H_2O, 90 ºC, 90 min	4.8%	9853	10148	1.03
	18.5%	5794	5881	1.01
	2.6%	1488	1504	1.01
	51.0%	980	992	1.01
	6.6%	573	578	1.01
**Lupranol® 2074**[a]	6.8%	1978	1980	1.00
	15.9%	1363	1365	1.00
	26.1%	1202	1205	1.00
	27.5%	962	966	1.00
	13.4%	761	764	1.00
	10.2%	359	361	1.01

[a] Because the pure polyol standard should not adsorb UV light, the species detected by PDA detector should be attributed to impurities, which have much lower molecular weight than the species detected by RI detector showed in Table S2.

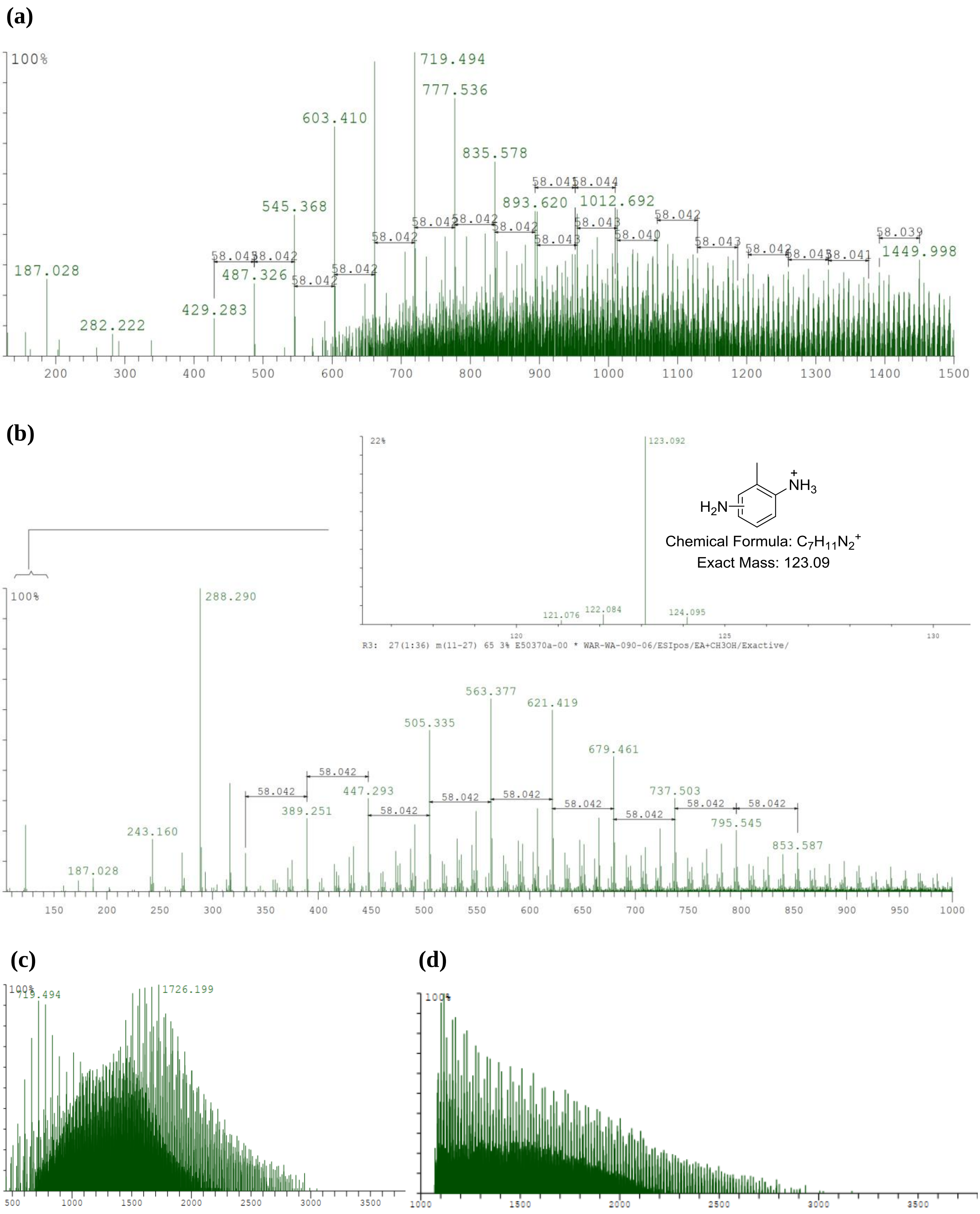

Figure S9. ESI-MS spectra of polyetherol standard (**a** and **c**, Lupranol® 2074) and the polyol (**b** and **d**) recovered from the hydrolysis of PU with Na_2CO_3 and 20 wt% Cu/$MgAlO_x$ via ball milling at 90 °C for 90 min (PO-3).

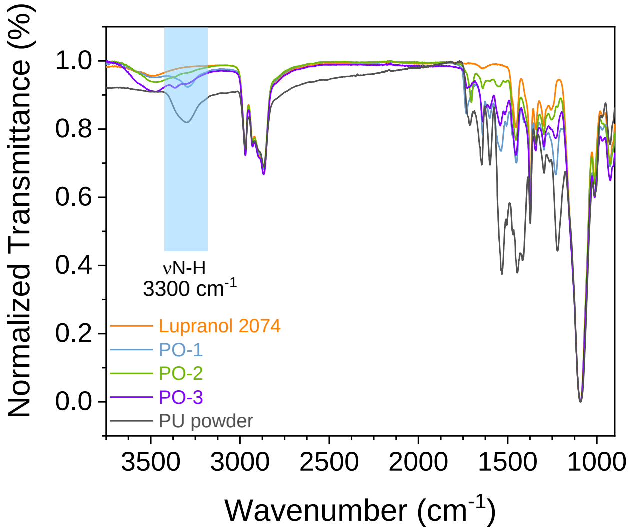

Figure S10. ATR-IR spectra (full range) of commercial polyetherol standard (Lupranol® 2074), initial PU powder and the polyols recycled from the PU powder.

Table S4. Elemental analysis results of the PU powder, commercial polyetherol standard (Lupranol® 2074) and recovered polyols.

Sample	% C	% H	% N	% O
PU powder	56.8	8.2	5.7	17.9
Lupranol® 2074	60.1	9.9	0.4	27.9
PO-1	59.4	9.7	2.7	26.3
PO-2	59.4	9.8	0.8	28.0
PO-3	58.8	9.7	1.8	27.6

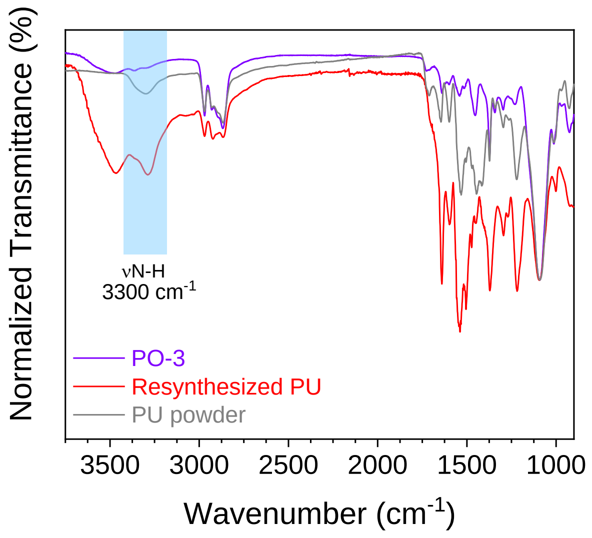

Figure S11. ATR-IR spectra (full range) of the recycled polyol, the insoluble PU resynthesized from the recycled polyol and TDI, and the initial PU powder.

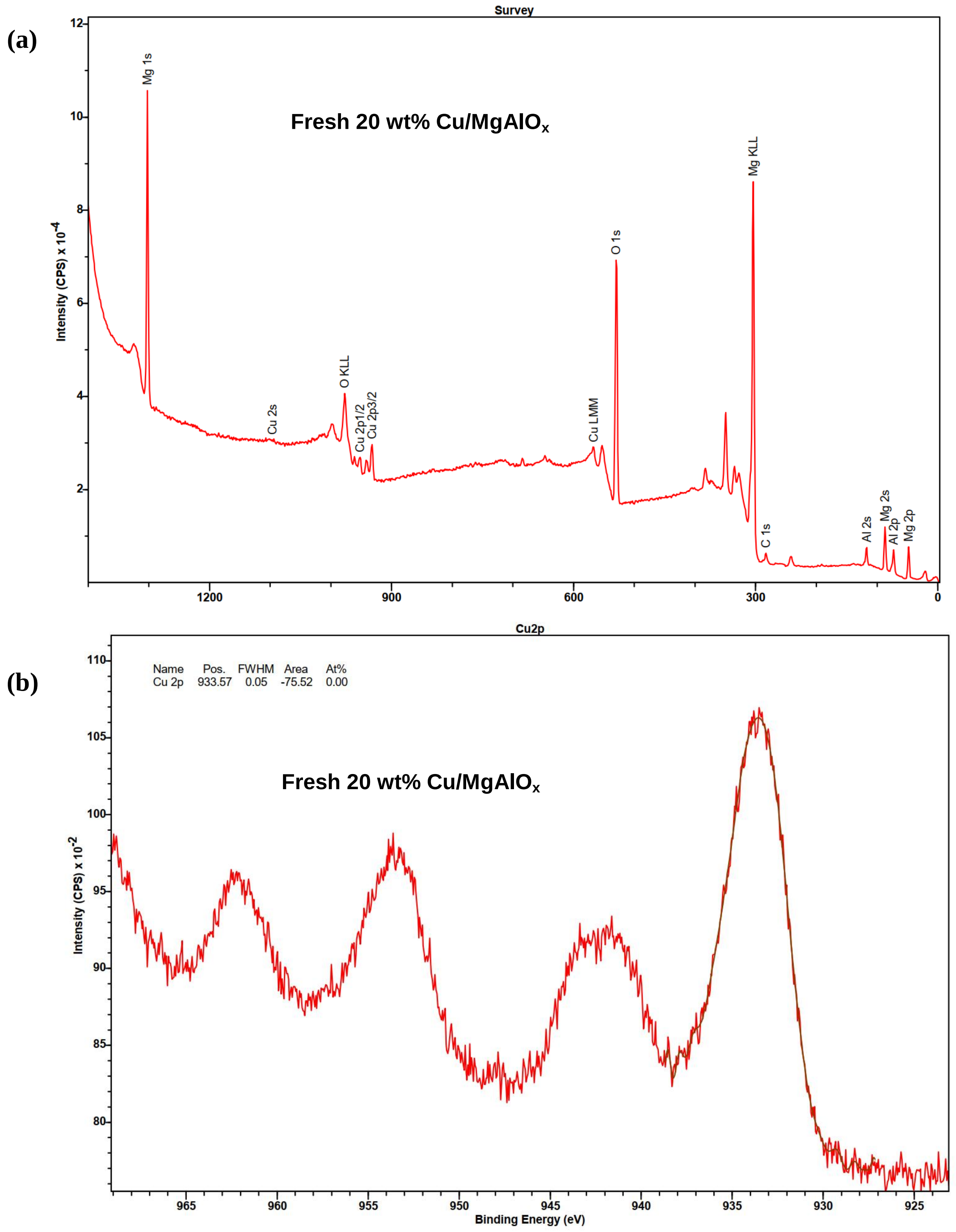

Figure S12. XPS spectra of fresh 20 wt% Cu/$MgAlO_x$. a) Survey spectrum. b) High-resolution scan spectrum over Cu 2p peaks.

(a)

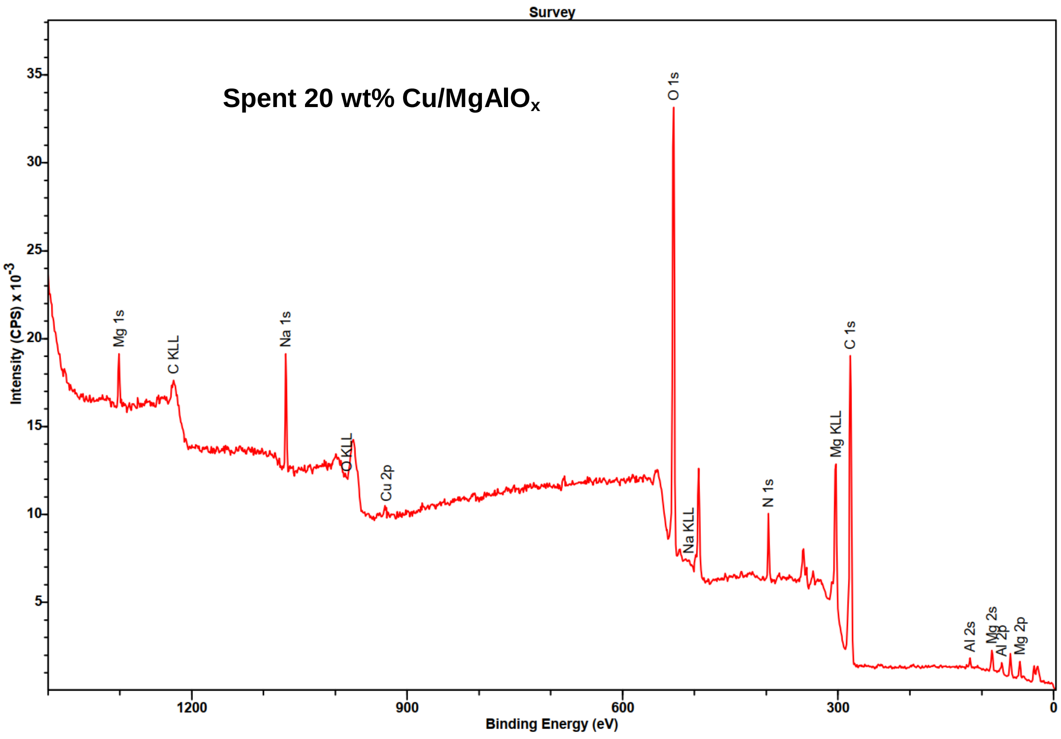
Survey
Spent 20 wt% Cu/MgAlOx
Intensity (CPS) x 10-3
Binding Energy (eV)
O 1s
Mg 1s
C KLL
Na 1s
O KLL
Cu 2p
Na KLL
N 1s
Mg KLL
C 1s
Al 2s
Mg 2s
Al 2p
Mg 2p

(b)

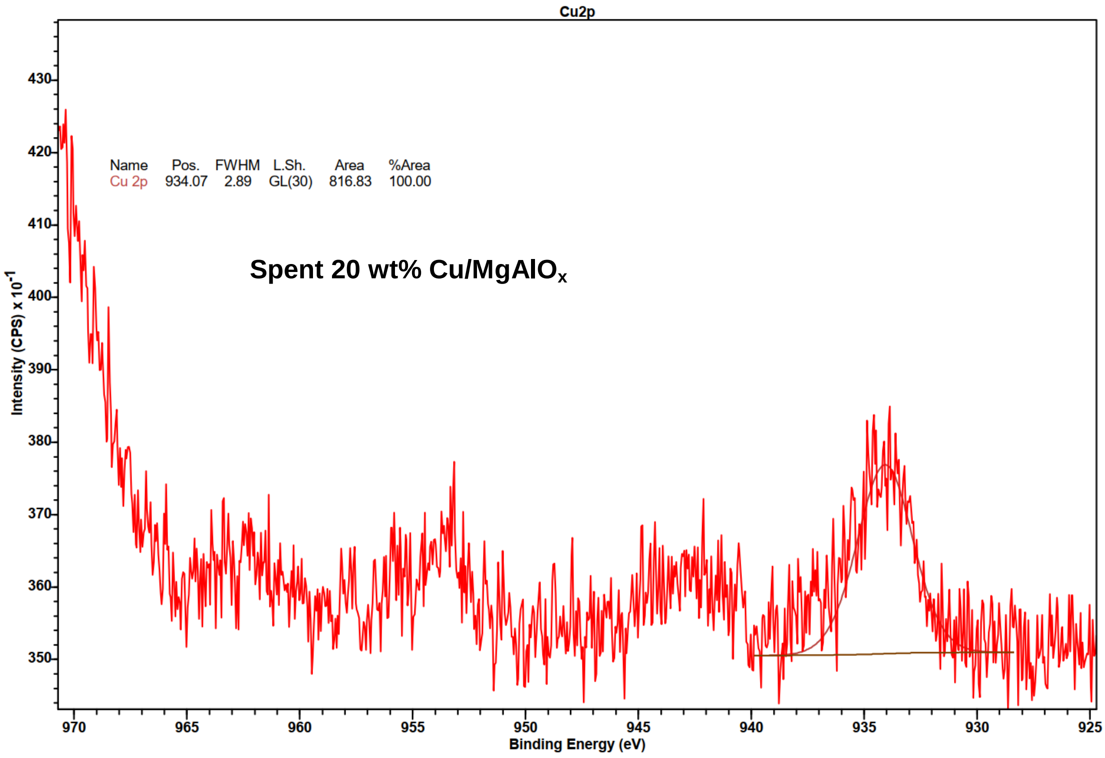
Cu2p
Name Pos. FWHM L.Sh. Area %Area
Cu 2p 934.07 2.89 GL(30) 816.83 100.00
Spent 20 wt% Cu/MgAlOx
Intensity (CPS) x 10-1
Binding Energy (eV)

(c)

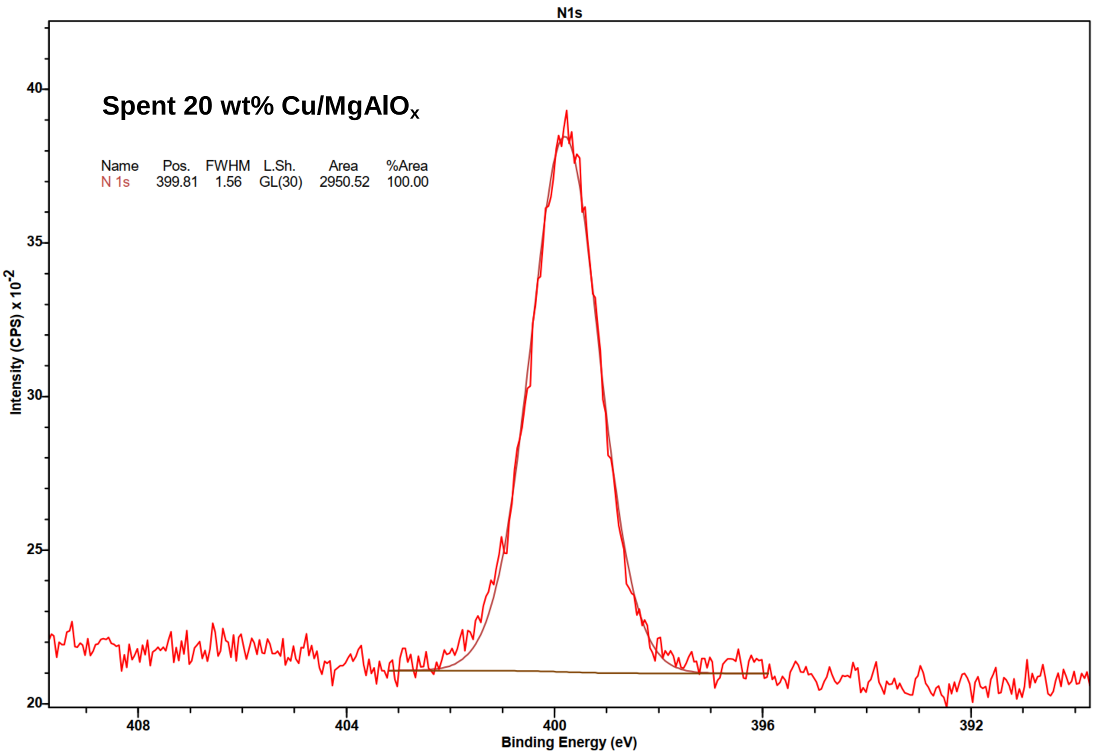

Figure S13. XPS spectra of spent 20 wt% Cu/MgAlO$_x$ recovered from the hydrolysis of 0.6 g PU catalyzed by Na_2CO_3. a) Survey spectrum and high-resolution scan spectrum over b) Cu 2p peaks and c) N 1s peaks

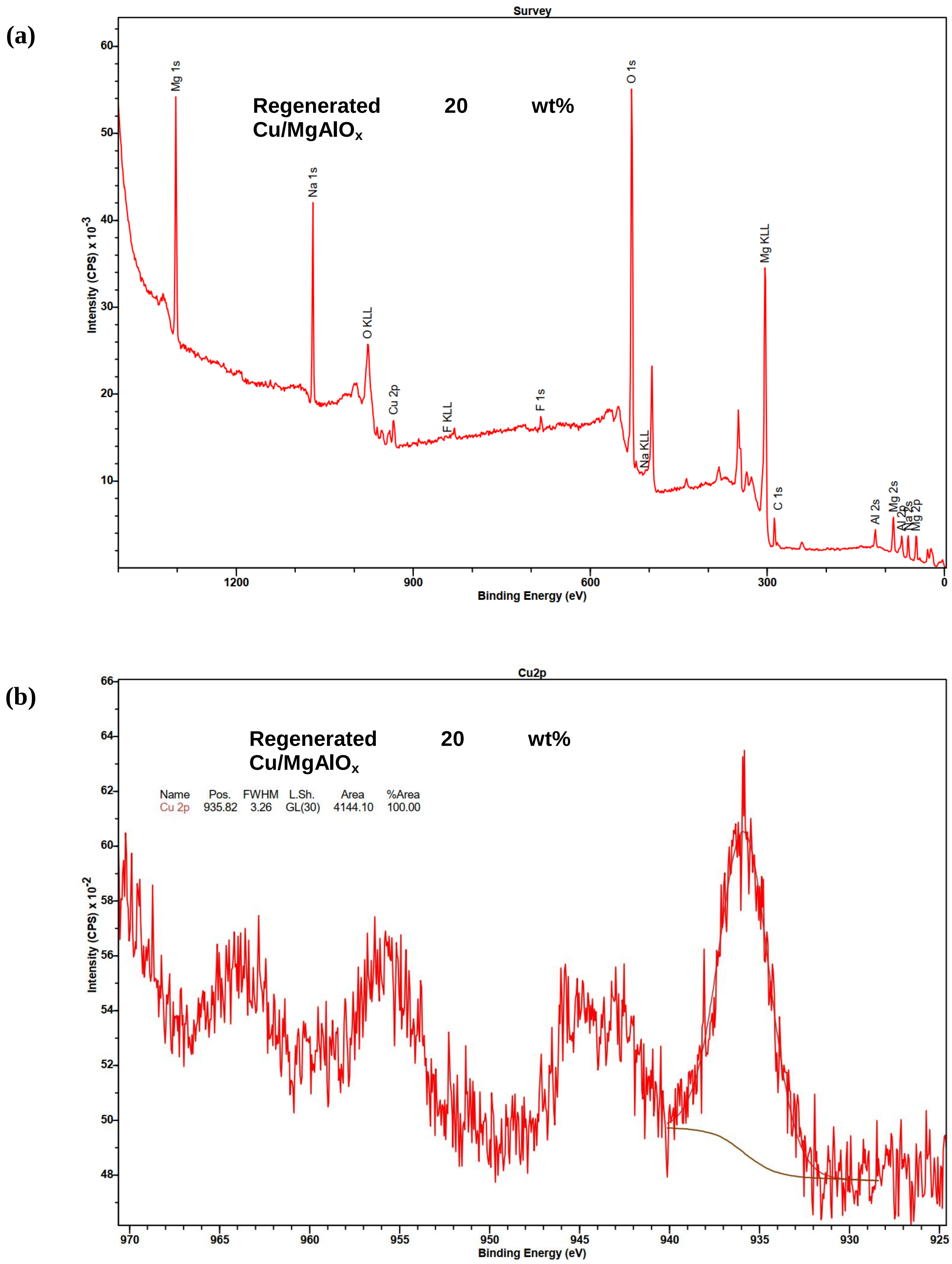

Figure S14. XPS spectra of regenerated 20 wt% Cu/$MgAlO_x$ calcinated in air at 550 °C for 5 hours. a) Survey spectrum and high-resolution scan spectrum over b) Cu 2p peaks.

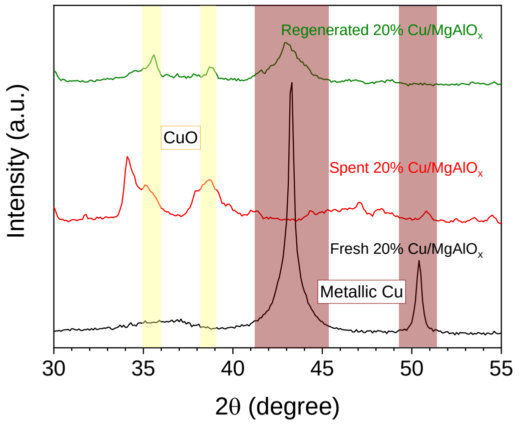

Figure S15. The XRD patterns of fresh, spent and regenerated 20 wt% Cu/MgAlO$_x$.

References

[1] W. Zhou, P. Neumann, M. Al Batal, F. Rominger, A. S. K. Hashmi, T. Schaub, *ChemSusChem* **2021**, *14*, 4176-4180.
