# Supplementary material for: Recycling of Polyurethane via Mechanocatalytic Methanolysis/Hydrolysis
Source: ChemSusChem. 2025 Apr 9;18(12):e202500253. doi: 10.1002/cssc.202500253 (PMC12175035; doi:10.1002/cssc.202500253)
Supplement: Supplementary file 1 — Supplementary Material [file CSSC-18-e202500253-s001.zip › cssc202500253-sup-0002-SuppData-S2.pdf]

PLASTICS EUROPE

Enabling a sustainable future

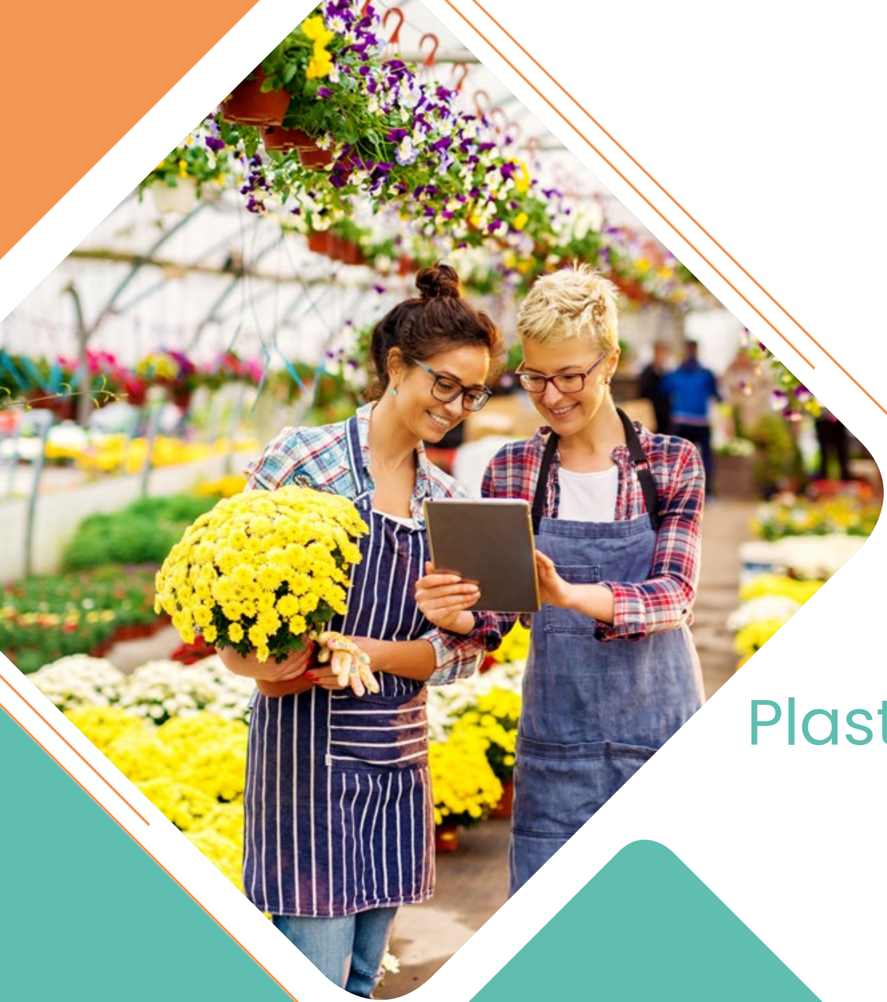

Plastics – the Facts 2021

An analysis of European plastics production, demand and waste data

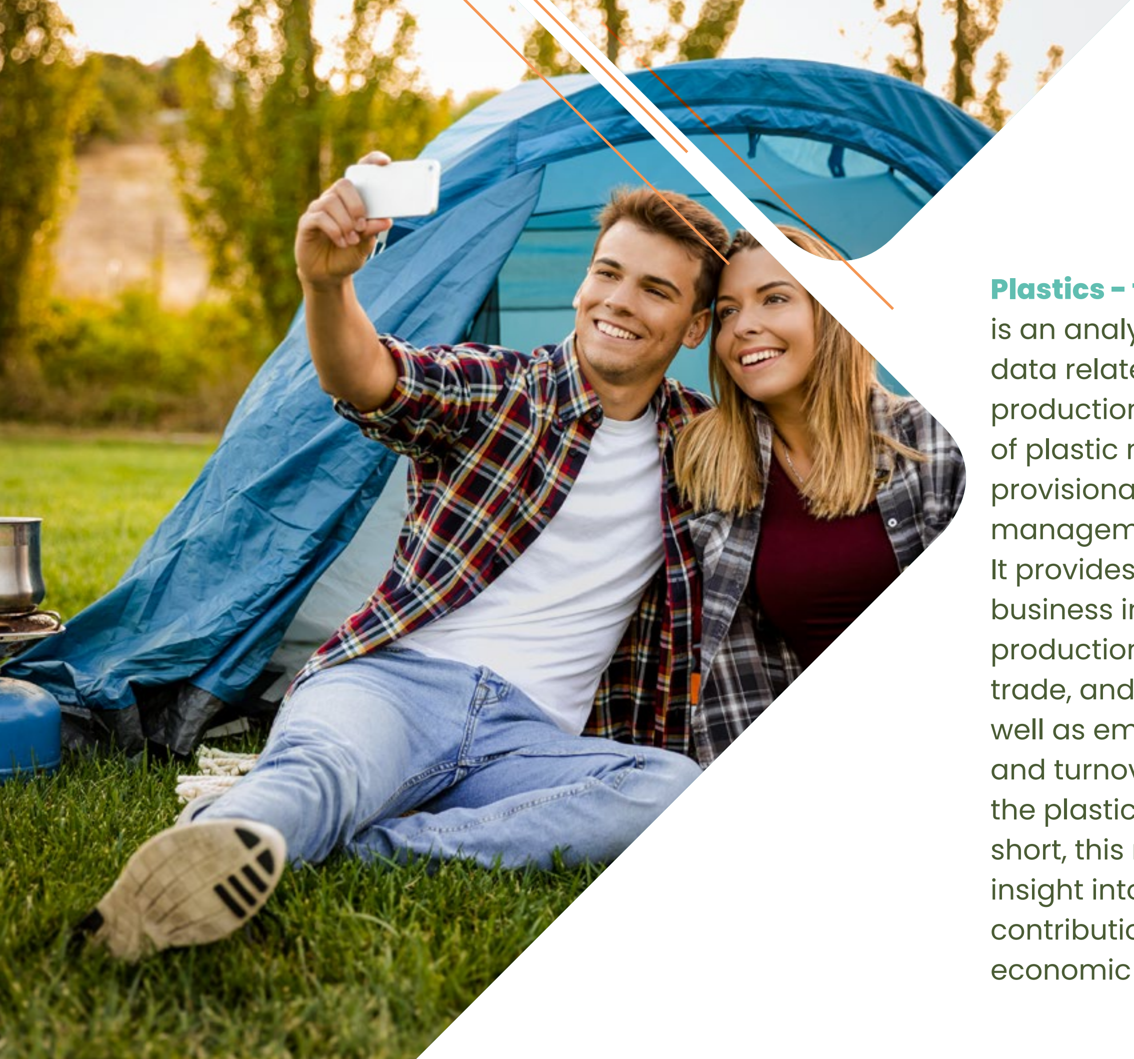

Plastics – the Facts
is an analysis of the data related to the production and demand of plastic materials and provisional plastic waste management data.
It provides the latest business information on production and demand, trade, and recovery as well as employment and turnover data in the plastics industry. In short, this report gives an insight into the industry's contribution to European economic growth.

The data presented in this report was collected by Plastics Europe (the Association of Plastics Manufacturers in Europe) and EPRO (the European Association of Plastics Recycling and Recovery Organisations).

Plastics Europe's Market Research Group (PEMRG) provided input on the production and demand of plastics. Conversio Market & Strategy GmbH helped assess waste collection and recovery data. Official statistics from European or national authorities and waste management organisations have been used for recovery and trade data, where available. Research or expertise from consultants completed gaps. Figures cannot always be directly compared with those of previous years due to changes in estimates.

Some estimates from previous years have been revised in order to track progress, e.g. for use and recovery of plastics across Europe over the past decade. All figures and graphs in this report show data for EU27 plus Norway, Switzerland and the United Kingdom, which is referred to as Europe for the purposes of abbreviation – other country groups are explicitly listed.

ENABLING
a sustainable future

Today, plastics deliver numerous benefits to society. They help feed the world in a safe and sustainable manner; they contribute to more energy efficient buildings and houses; they allow great fuel savings in all transportation means ensuring the transition to a green mobility, and they can even save our lives.

Undoubtedly, plastics are key materials in innovation and in reducing energy demand while reducing green gas emissions. And as essential materials for society, our sector must ensure that plastics are sustainable and have a positive impact on people and on the planet.

The plastics industry supports the European Union's Green Deal and climate-neutrality ambitions, and the Paris Climate Agreement - our collective blueprint for accelerating the transformation to a more sustainable Europe. Our committment as an industry is to relentlessly focus on ensuring plastics continue to enable and deliver benefits valued by society, while minimising their environmental footprint.

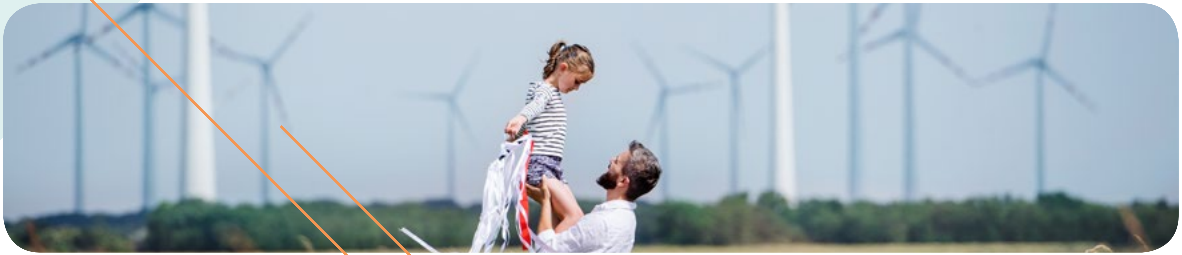

The current report is an analysis of plastics EU trade data, virgin plastics production, virgin plastics converters demand and preliminary post-consumer plastic waste management in the EU27+3 in 2020. This report does not show data related to the production and use of recycled plastics.

For a more exhaustive analysis of plastics progress towards circularity in Europe, including more in-depth post-consumer plastic waste management figures; information on the production and uptake of recycled plastics in new products; and the different available technologies to turn plastic waste into new resources, please refer to the report **"The Circular Economy of Plastics – A European Overview"**. An update of this report will be released early 2022 by Plastics Europe.

Despite the unexpected challenging circumstances, in 2020, the European plastics industry rapidly adapted its capacities to continue delivering safe and sustainable solutions to society.

As a consequence of the COVID crisis, in 2020, the European plastics value-chain, composed by plastics producers, plastics converters, plastics recyclers and machinery manufacturers, experienced a decrease both in its production and demand levels. Nevertheless, this sector was still able to maintain a high level of employment. With close to 1.5 million people working in over 50,000 companies, most of them SMEs distributed all over Europe, the plastics sector is key to the European economic recovery.

Regarding end-of-life management, the industry increased its efforts to accelerate the circularity of plastics and, although the overall European recycling activities faced serious difficulties, especially in the second quarter of 2020, the plastics post-consumer waste recycling rate increased and the quantities sent to energy recovery processes remained stable and to landfill decreased.

1

CONTRIBUTION to European society

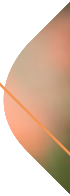

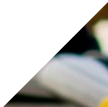

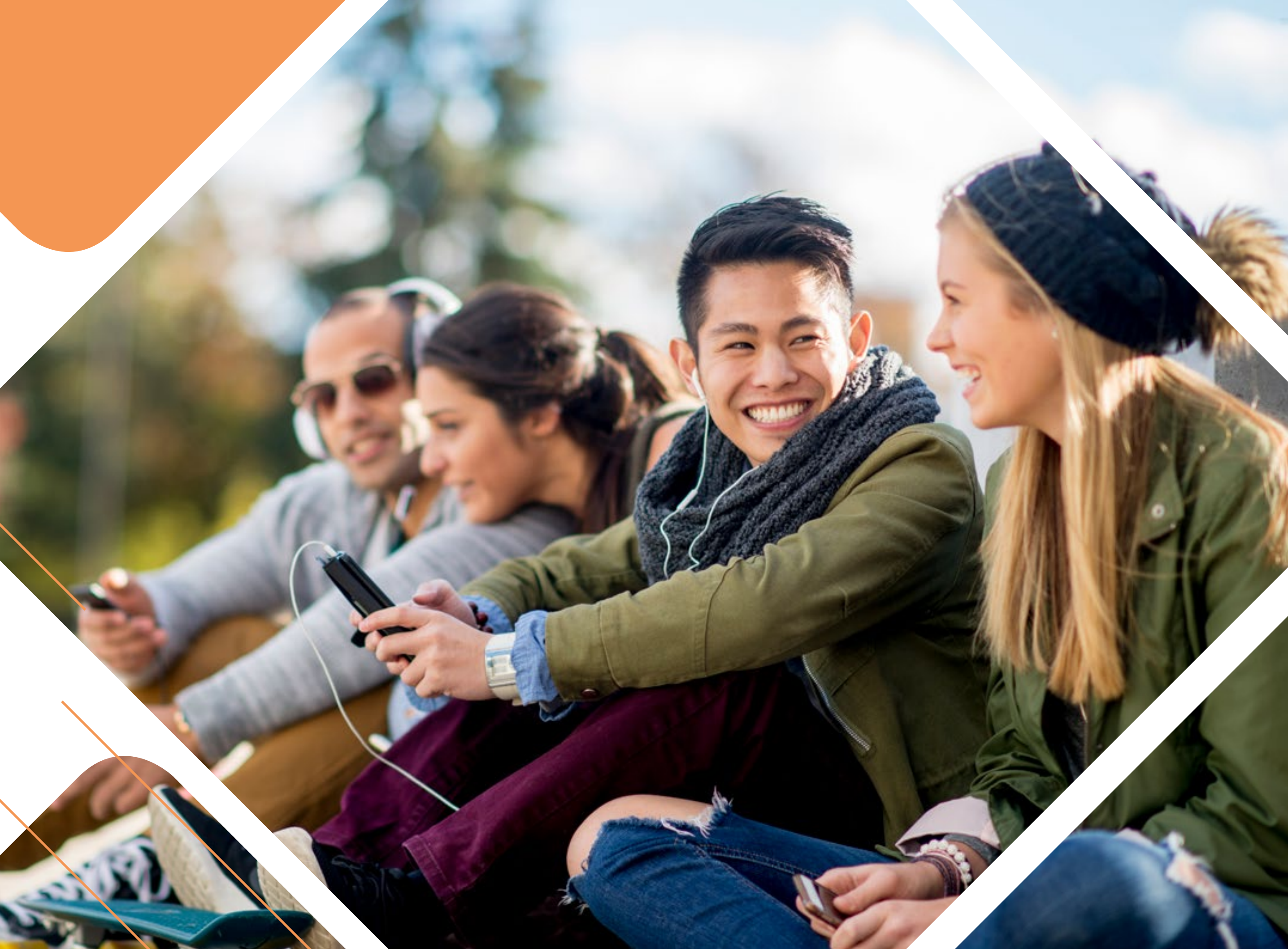

The European plastics industry:

KEY FIGURES*

The European plastics industry includes plastics producers, plastics converters, plastics recyclers and plastics & rubber machinery producers in the EU27+3.

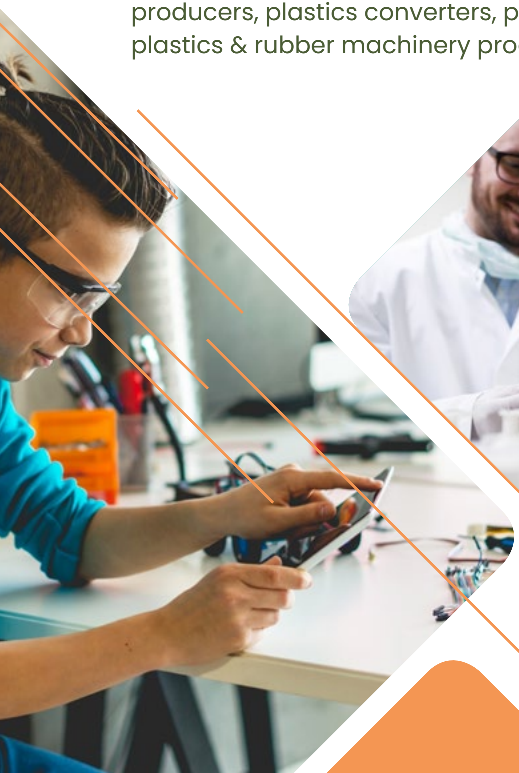

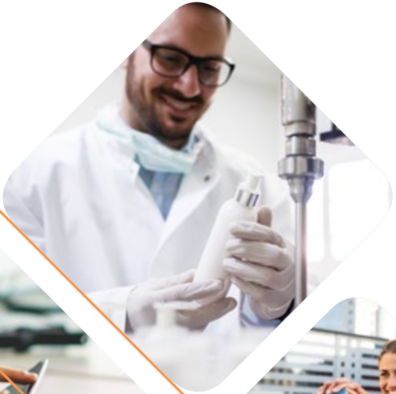

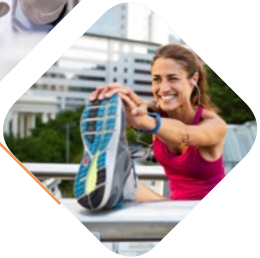

EMPLOYEMENT

CLOSE TO 1.5 MILLION

In 2020, the European plastics industry maintained its level of employment with a brief slowdown compared to 2019.

COMPANIES

CLOSE TO 52,000

In 2020, the number of companies slightly decreased but still remained over 50,000 – demonstrating the contribution of this sector to the European industrial fabric.

TURNOVER

CLOSE TO EUR 330 BILLION €

In 2020, the turnover of the European plastic industry showed a slight decrease compared to the previous year, mainly due to the impact of the COVID-19 crisis on the majority of customer industries.

KEY FIGURES

* 2020 Plastics Europe estimations – Eurostat official data only available until 2018.

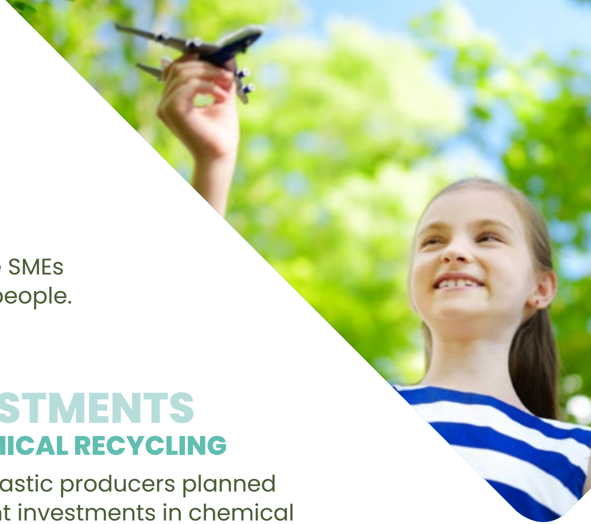

EMPLOYEES PER COMPANY

LESS THAN 30

The vast majority of companies in the plastics sector in Europe are SMEs as the average number of employees per company is around 29 people.

INDUSTRIAL VALUE ADDED

8TH MOST IMPORTANT INDUSTRY*

The European plastics industry ranks 8th in Europe in industrial value-added contribution. It stands at a similar level with the electrical equipment and close to the pharmaceutical industry.

INVESTMENTS

IN CHEMICAL RECYCLING

In 2021, plastic producers planned significant investments in chemical recycling technologies – ramping up from EUR 2.6 billion in 2025 to EUR 7.2 billion in 2030.

RECYCLING

10,2 Mt

In 2020, almost 10.2 million tonnes of post-consumer plastic waste were collected and sent to recycling facilities inside and outside Europe.

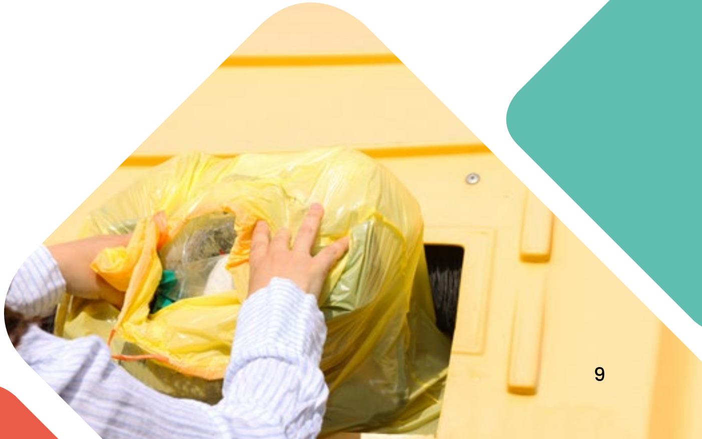

INDUSTRIAL VALUE ADDED

* Measured by value added at factor cost, 2018.

2 PRODUCTION and trade

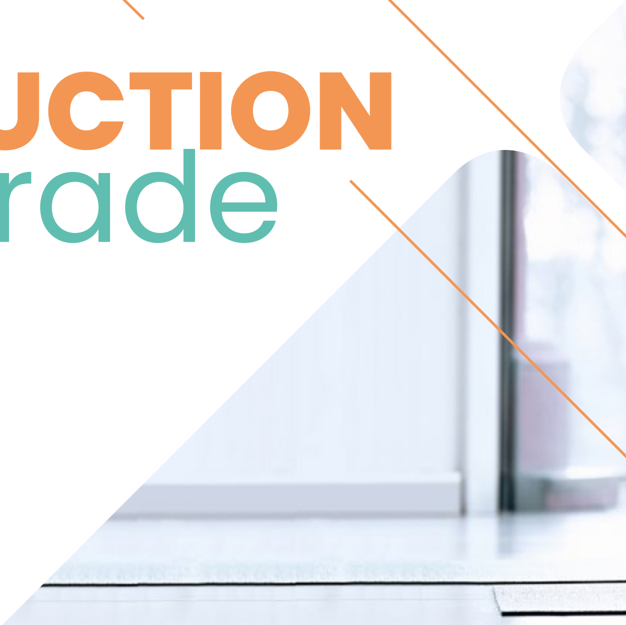

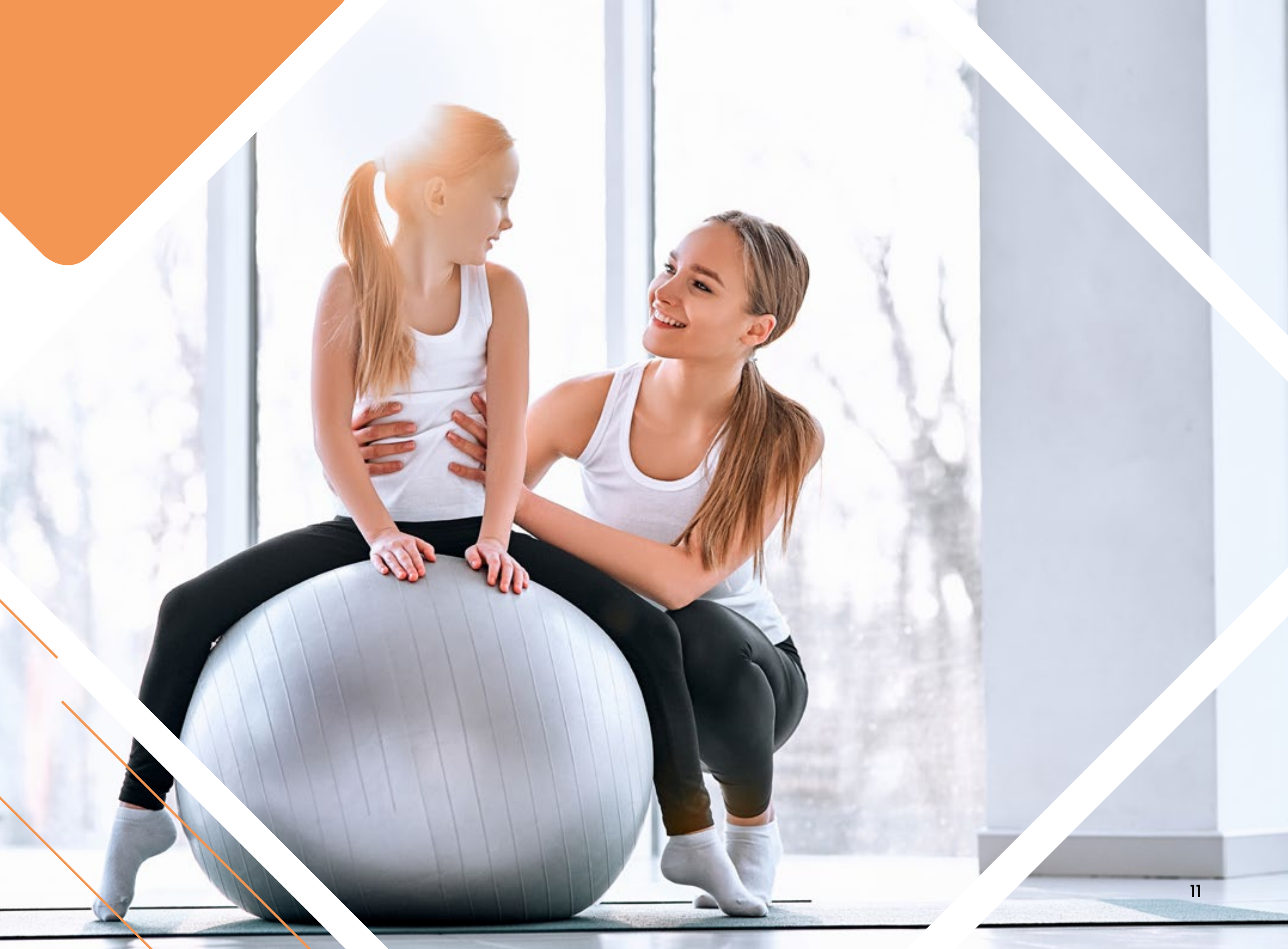

WORLD AND EUROPEAN
plastics production evolution

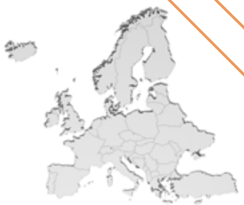

WORLD PLASTICS PRODUCTION

These figures do not include the production of recycled plastics

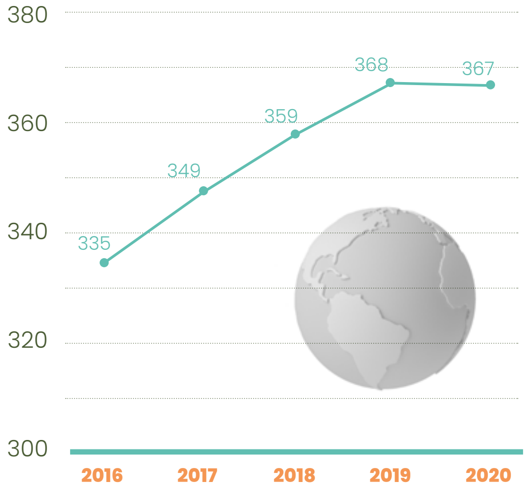

EUROPEAN PLASTICS PRODUCTION

These figures do not include the production of recycled plastics

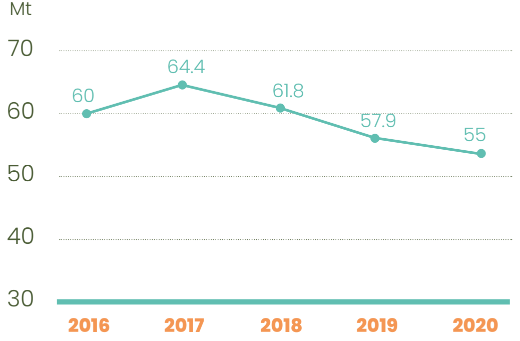

Includes Thermoplastics, Polyurethanes, Thermosets, Elastomers, Adhesives, Coatings and Sealants and PP-Fibers. Not included PET-, PA- and Polyacryl-Fibers.

SOURCE: Plastics Europe Market Research Group (PEMRG) and Conversio Market & Strategy GmbH.

Estimated data.

DISTRIBUTION

of the global plastics production

**CHINA**	32%
**NAFTA***	19%
**REST OF ASIA**	17%
**EUROPE**	15%
**MIDDLE EAST, AFRICA**	7%
**LATIN AMERICA**	4%
**JAPAN**	3%
**CIS****	3%

367 Mt***

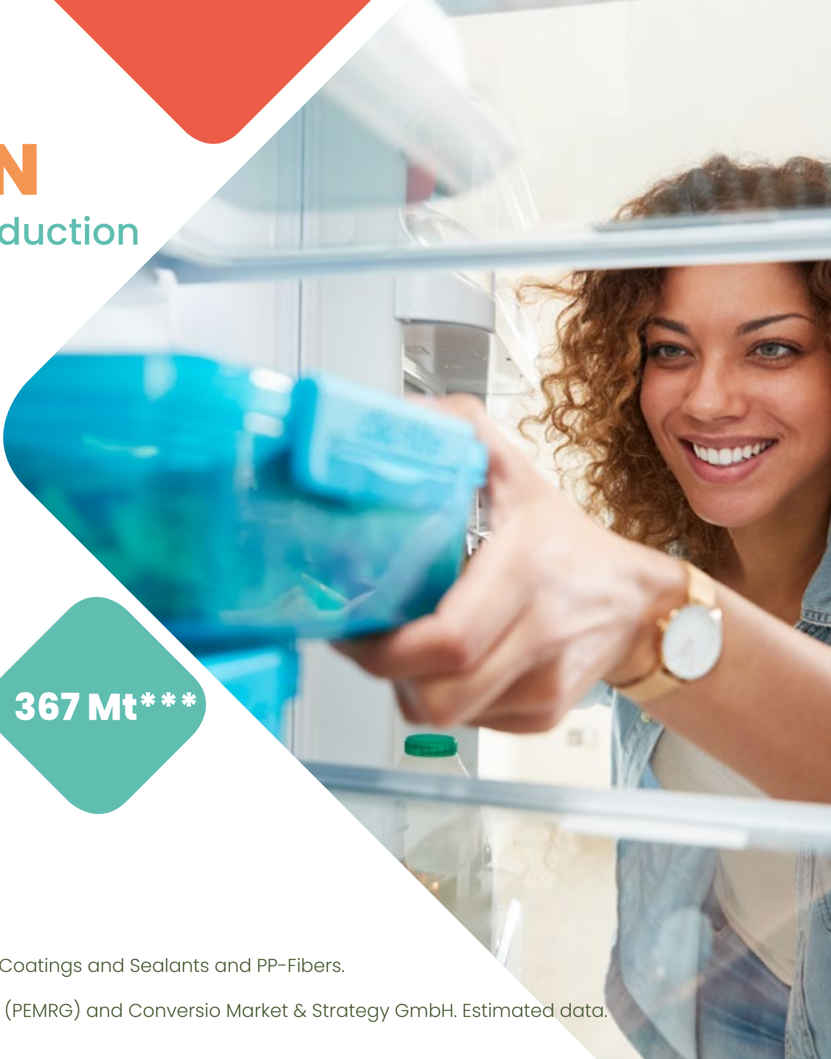

* North American Free Trade Agreement.

** Commonwealth of Independent States.

*** Includes Thermosets, Elastomers, Adhesives, Coatings and Sealants and PP-Fibers.
Not included PET-, PA- and Polyacryl-Fibers.

SOURCE: Plastics Europe Market Research Group (PEMRG) and Conversio Market & Strategy GmbH. Estimated data.

TRADE BALANCE

Close to EUR 16 billion in 2020

In 2020, the European plastics industry achieved a positive trade balance of EUR 15.8 billion.

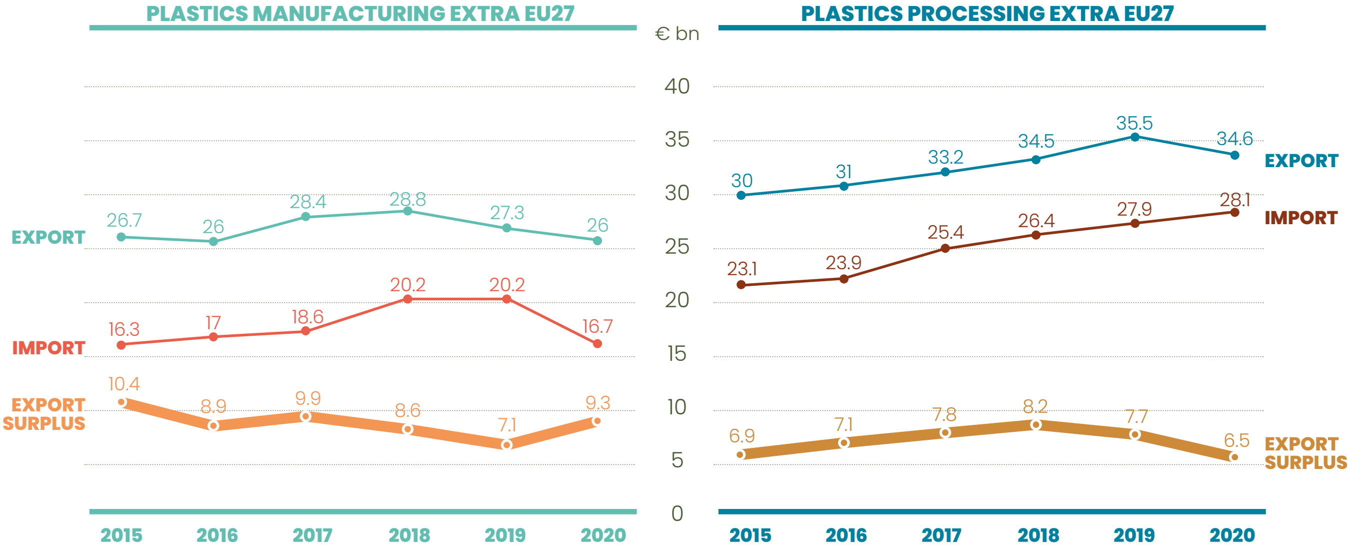

SOURCE: Eurostat.

TOP EXTRA EU TRADE PARTNERS

in value

In 2020, the USA, United Kingdom and China were the top trade partners of the EU27 plastics industry.

PLASTICS MANUFACTURING IMPORTS

USA	22.8%
UNITED KINGDOM	14.3%
SOUTH KOREA	11%
SAUDI ARABIA	8.6%
CHINA	6.4%

PLASTICS MANUFACTURING EXPORTS

UNITED KINGDOM	15%
CHINA	12.7%
TURKEY	12.3%
USA	10%
RUSSIA	6%

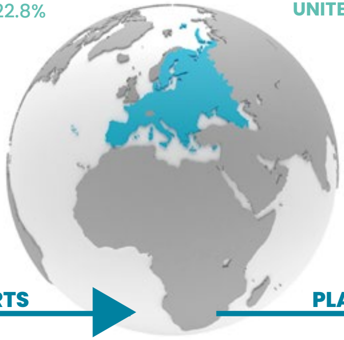

PLASTICS PROCESSING IMPORTS

CHINA	33.8%
USA	11.7%
SWITZERLAND	7.8%
TURKEY	6.8%
SOUTH KOREA	3.3%

PLASTICS PROCESSING EXPORTS

UNITED KINGDOM	18.4%
USA	12.9%
SWITZERLAND	9.5%
CHINA	7.8%
RUSSIA	5.9%

SOURCE: Eurostat.

3 MARKET demand

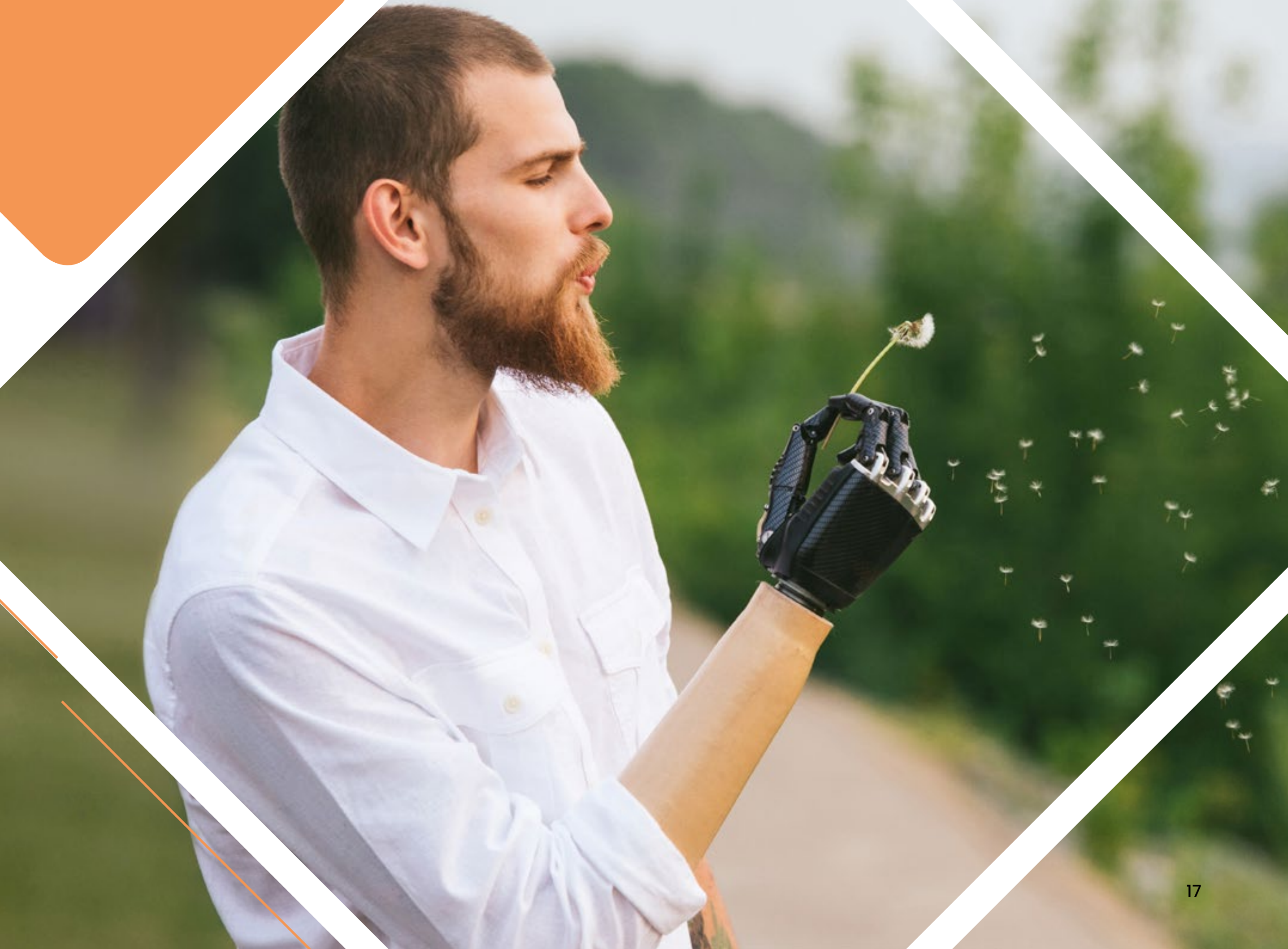

Converters plastics demand
BY COUNTRIES

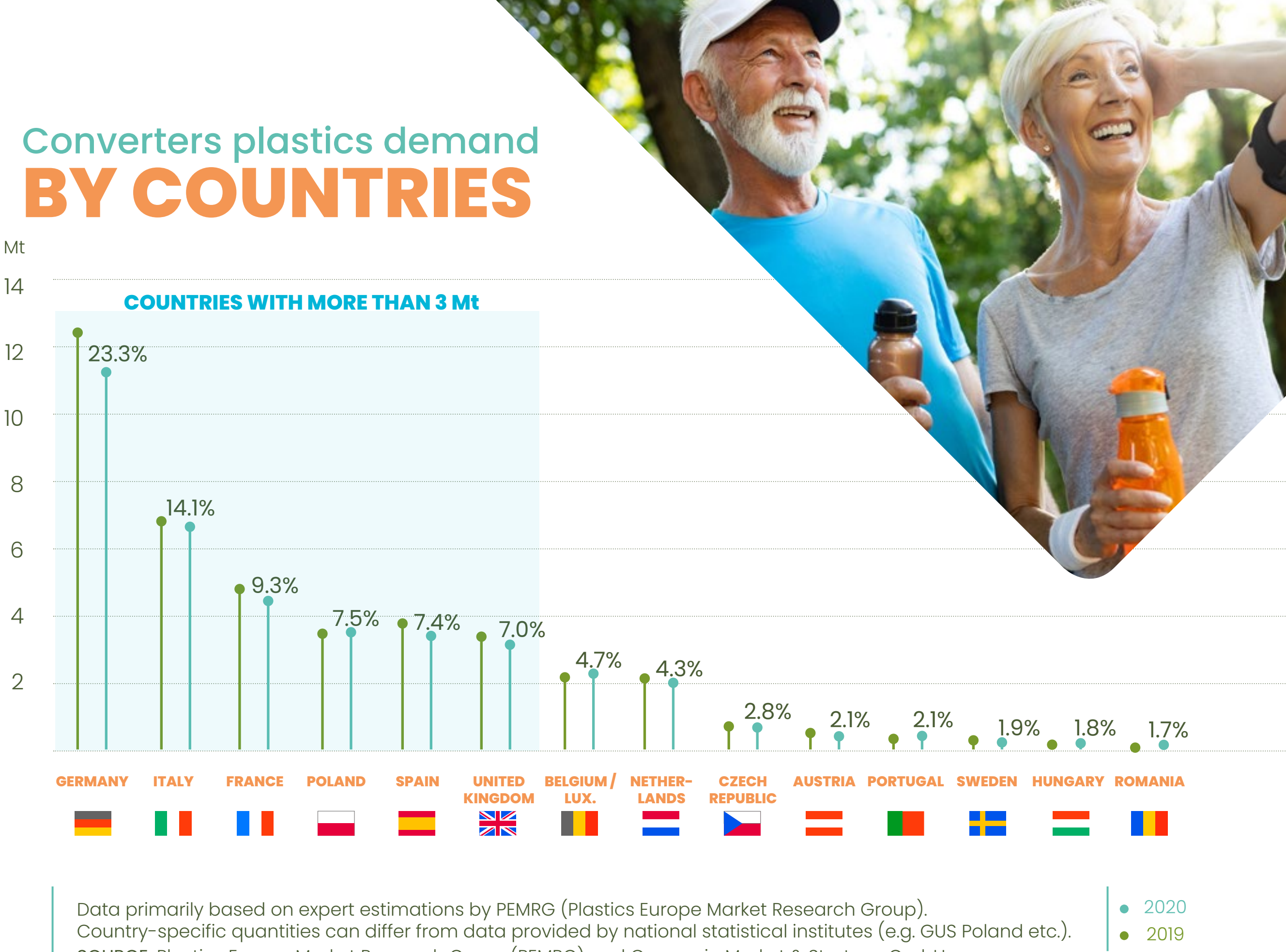

Data primarily based on expert estimations by PEMRG (Plastics Europe Market Research Group).
Country-specific quantities can differ from data provided by national statistical institutes (e.g. GUS Poland etc.).
SOURCE: Plastics Europe Market Research Group (PEMRG) and Conversio Market & Strategy GmbH.
Demand estimations do not include recycled plastics.

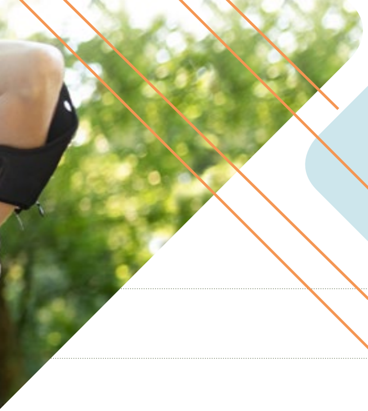

CONVERTERS PLASTICS DEMAND IN EU27+3

49.1 Mt

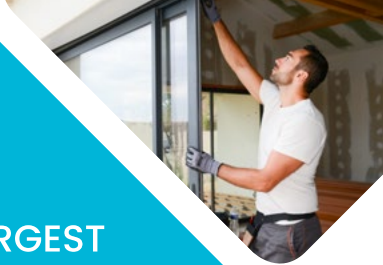

THE 6 LARGEST EUROPEAN COUNTRIES REPRESENT ALMOST

70%

OF THE MARKET DEMAND

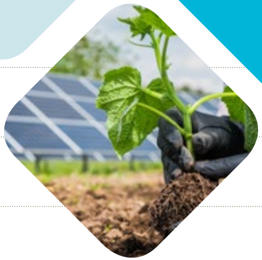

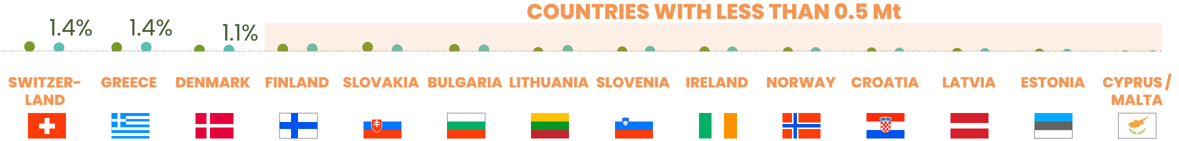

Data primarily based on expert estimations by PEMRG (Plastics Europe Market Research Group).
Country-specific quantities can differ from data provided by national statistical institutes (e.g. GUS Poland etc.).
SOURCE: Plastics Europe Market Research Group (PEMRG) and Conversio Market & Strategy GmbH.
Demand estimations do not include recycled plastics.

EU27+3 converters plastics demand

BY SEGMENTS 2020

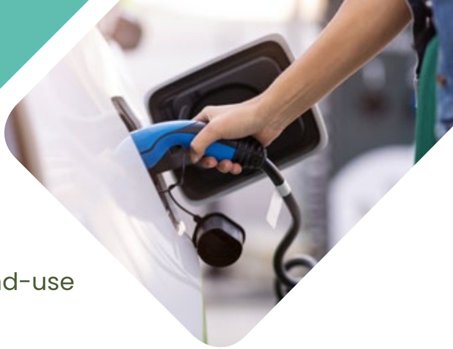

Packaging and Building & Construction by far represent the largest end-use markets.

The third biggest end-use market is the **Automotive Industry**.

"Others" includes plastics for furniture, medical applications, machinery and mechanical engineering, technical parts etc.

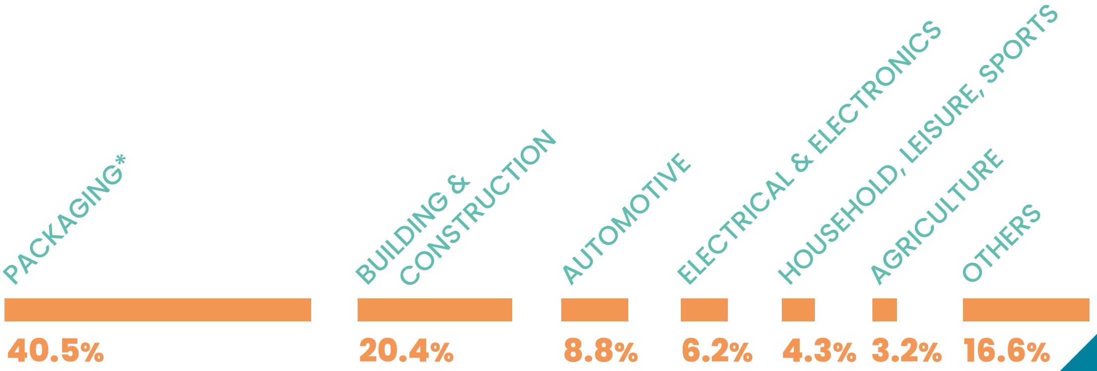

Total 49.1 Mt

* Including commercial and industrial packaging.

SOURCE: Plastics Europe Market Research Group (PEMRG) and Conversio Market & Strategy GmbH.

Demand estimations do not include recycled plastics.

EU27+3 converters plastics demand
BY POLYMER TYPES 2020

PLASTICS DEMAND IN EU27+3
49.1 Mt

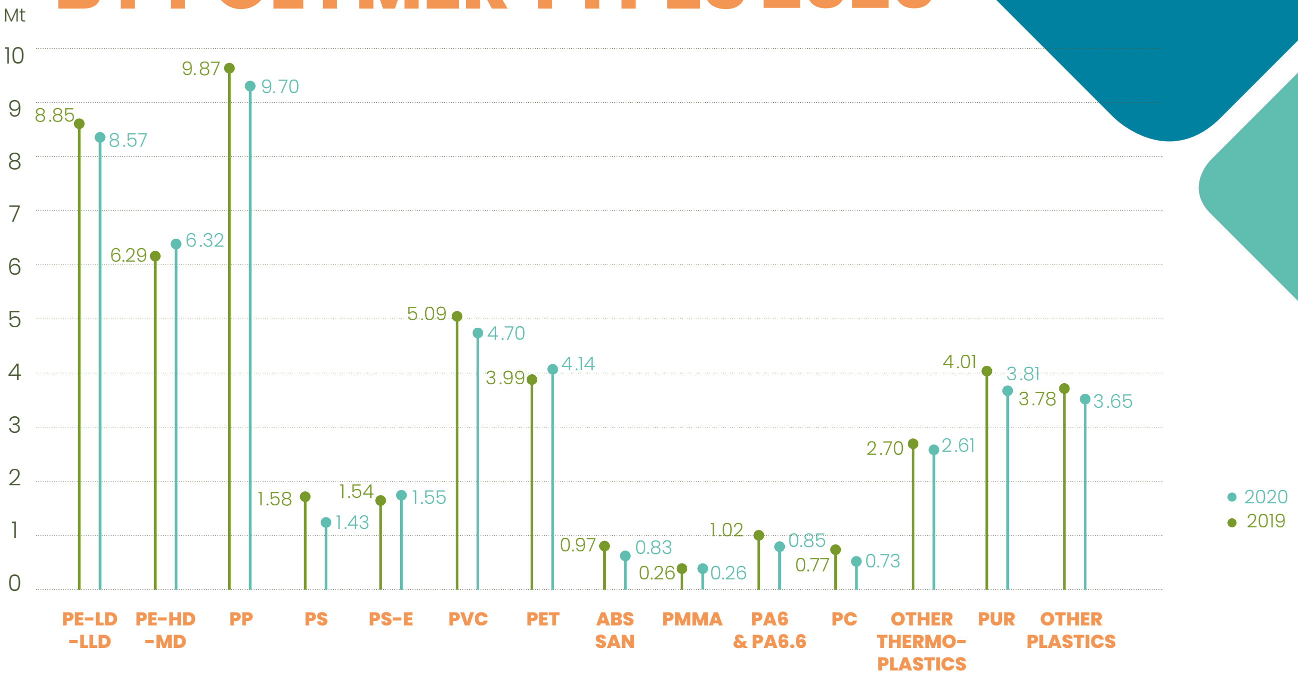

SOURCE: Plastics Europe Market Research Group (PEMRG) and Conversio Market & Strategy GmbH.
Demand estimations do not include recycled plastics.

EU27+3 converters plastics demand

DISTRIBUTION BY POLYMER TYPES 2020

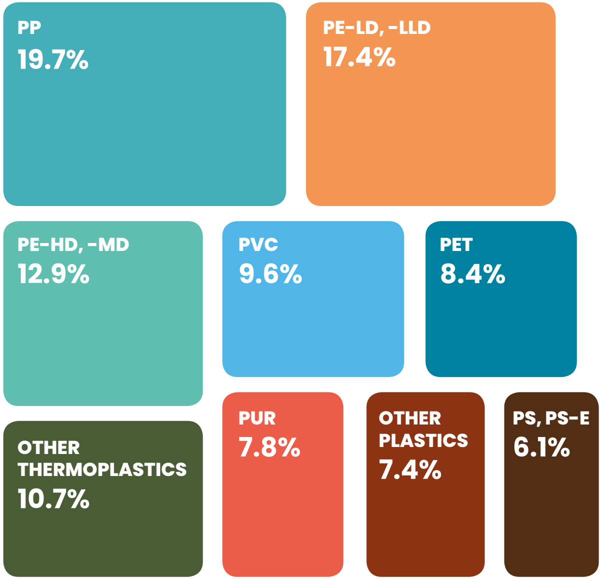

PP | Food packaging, sweet and snack wrappers, hinged caps, microwave containers, pipes, automotive parts, bank notes, etc.

PE-LD, -LLD | Reusable bags, trays and containers, agricultural film, food packaging film, etc.

PE-HD, -MD | Toys, milk bottles, shampoo bottles, pipes, houseware, etc.

PVC | Window frames, profiles, floor and wall covering, pipes, cable insulation, garden hoses, inflatable pools, etc.

PET | Bottles for water, soft drinks, juices, cleaners, etc.

PUR | Building insulation, pillows and mattresses, insulating foams for fridges, etc.

OTHER PLASTICS | Includes other thermosets such as phenolic resins, epoxide resins, melamine resins, urea resins and others.

PS, PS-E | Food packaging (dairy, fishery), building insulation, electrical & electronic equipment, inner liner for fridges, eyeglasses frames, etc.

OTHER THERMOPLASTICS | Hub caps (ABS); optical fibres (PBT); eyeglasses lenses, roofing sheets (PC); touch screens (PMMA); cable coating in telecommunications (PTFE); and many others in aerospace, medical implants, surgical devices, membranes, valves & seals, protective coatings, etc.

SOURCE: Plastics Europe Market Research Group (PEMRG) and Conversio Market & Strategy GmbH.
Demand estimations do not include recycled plastics.

Total:
49.1 Mt

EU27+3 converters plastics demand

BY SEGMENTS & POLYMER 2020

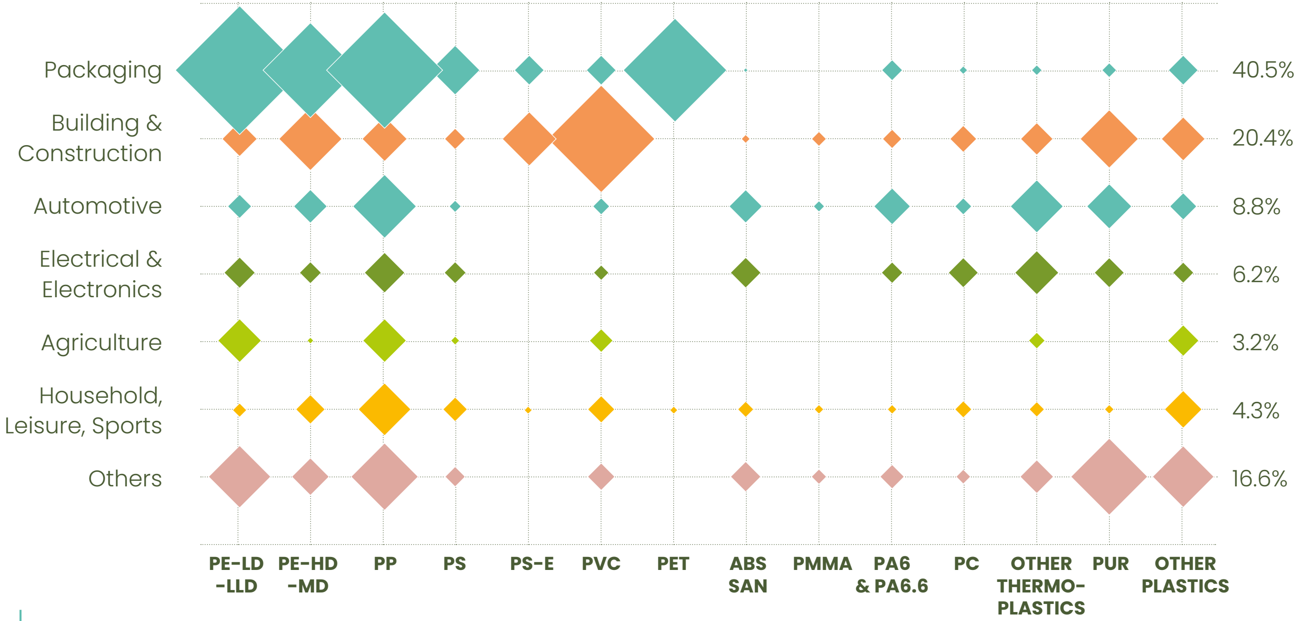

SOURCE: Plastics Europe Market Research Group (PEMRG) and Conversio Market & Strategy GmbH.
Demand estimations do not include recycled plastics.
Numbers behind this graph are available upon request.

4

END-OF-LIFE management

Preliminary data

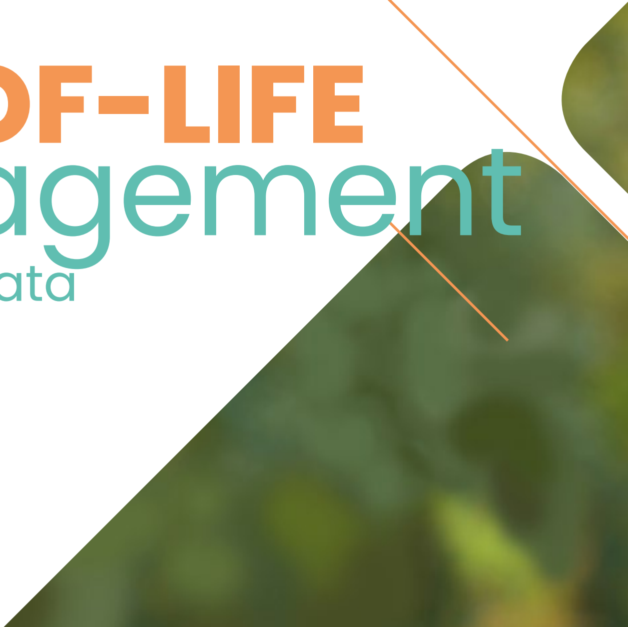

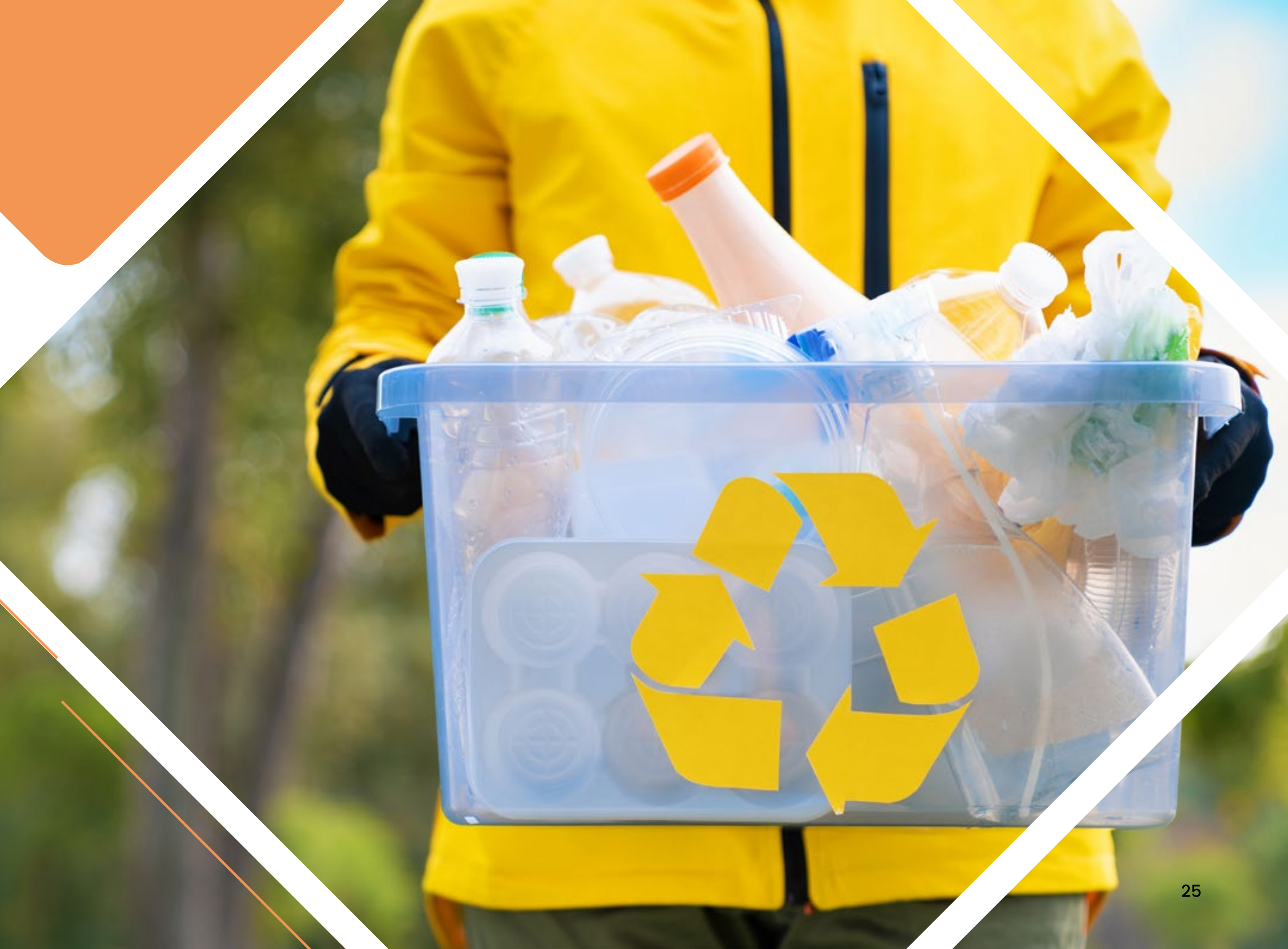

POST-CONSUMER PLASTIC WASTE

treatment in 2020 (preliminary data)

In 2020, more than 29 million tonnes of plastic post-consumer waste were collected in the EU27+3. Because plastics products have different life span (ranginging from 1 to 50 years or more), of post-consumer plastic waste collection figures do not match demand or consumption figures.

More than **one third was sent to recycling facilities inside and outside the EU27+3** but over 23% was still sent to landfill and more than 40% was sent to energy recovery operations.

29.5 Mt
Collected plastic post-consumer waste

-16 %
Extra-EU plastic waste exports

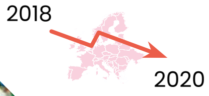

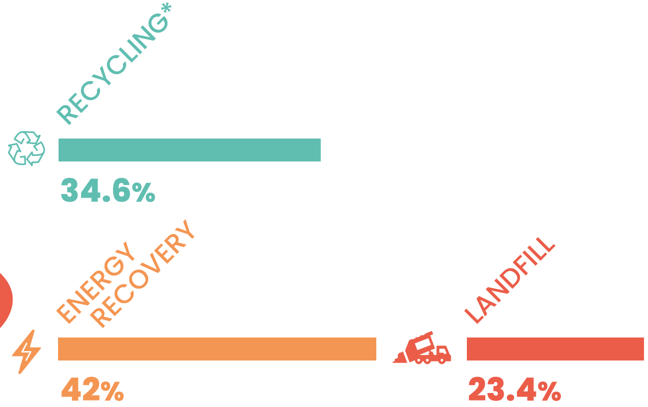

*** RECYCLING**: including 0.2% from chemical recycling.
SOURCE: Conversio Market & Strategy GmbH
Above data are rounded estimations based on extrapolations of 2019 waste data for 2020.

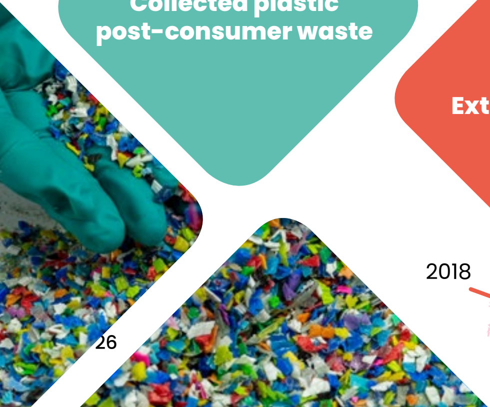

PLASTIC POST-CONSUMER WASTE

treatment in 2020 (preliminary data)

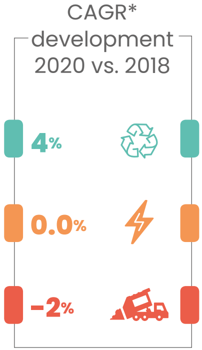

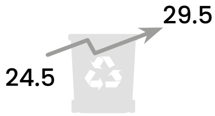

2006—2020
Total waste collected
(Mt)

2006-2020 evolution of post-consumer plastic waste treatment in EU27+3

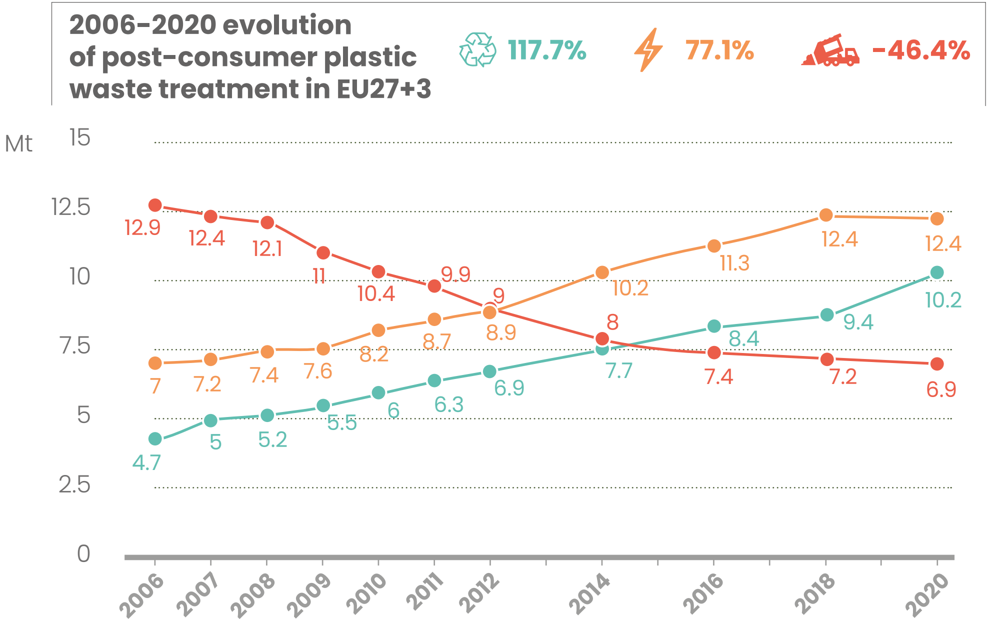

* **CAGR:** Compound Annual Growth Rate is the mean annual growth rate over a specific period of time.
SOURCE: Conversio Market & Strategy GmbH.
Above data are rounded estimations based on extrapolations of 2019 waste data for 2020.

5 OUTLOOKS

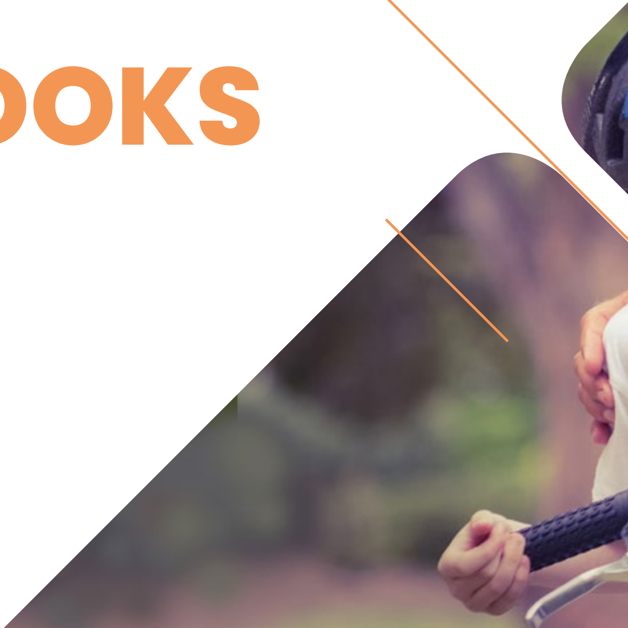

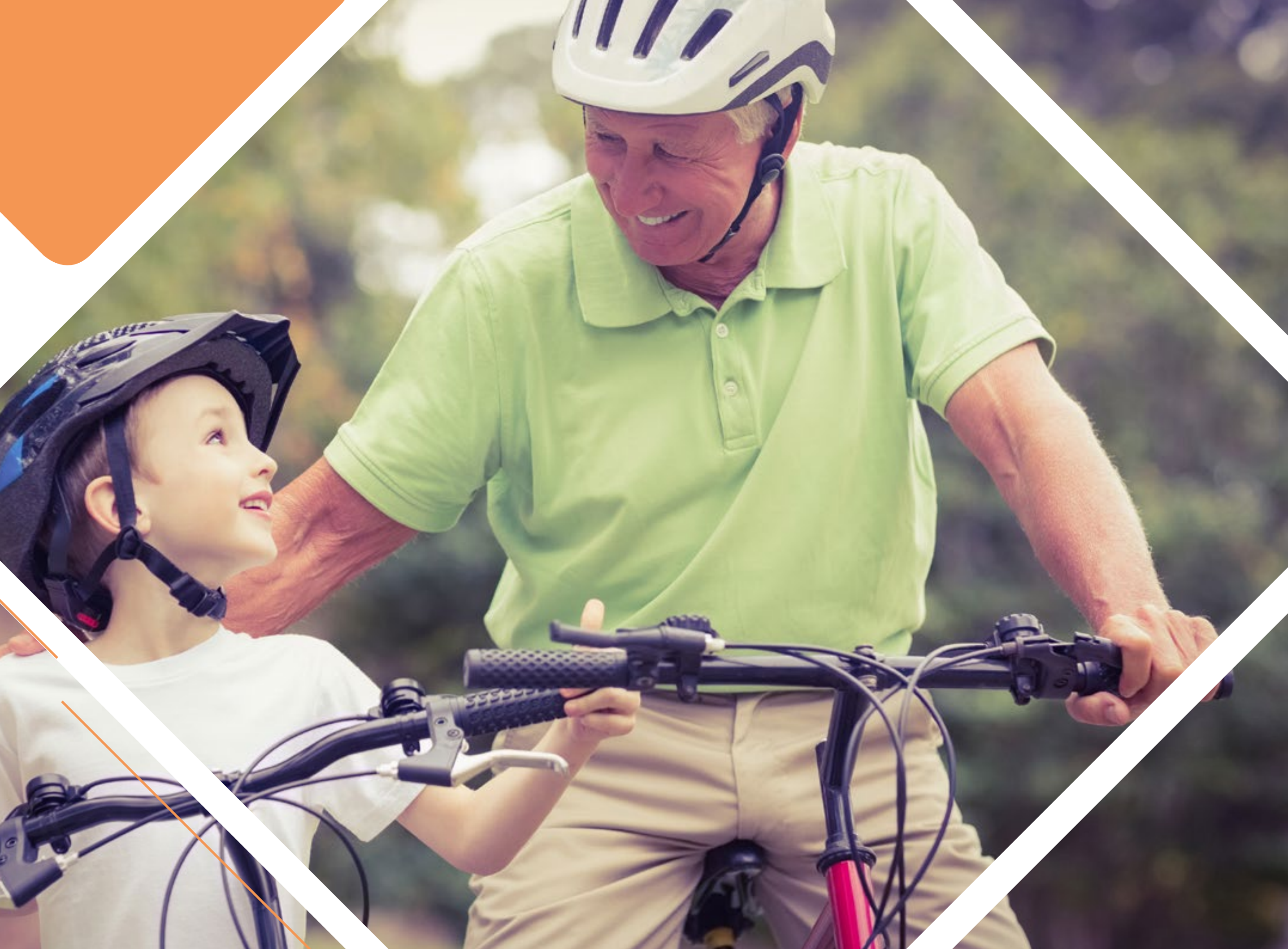

PLASTICS INDUSTRY PRODUCTION

in EU27

The sharp decline in production for the European plastics industry due to the coronavirus pandemic in the first half of 2020 was followed by an even stronger recovery.

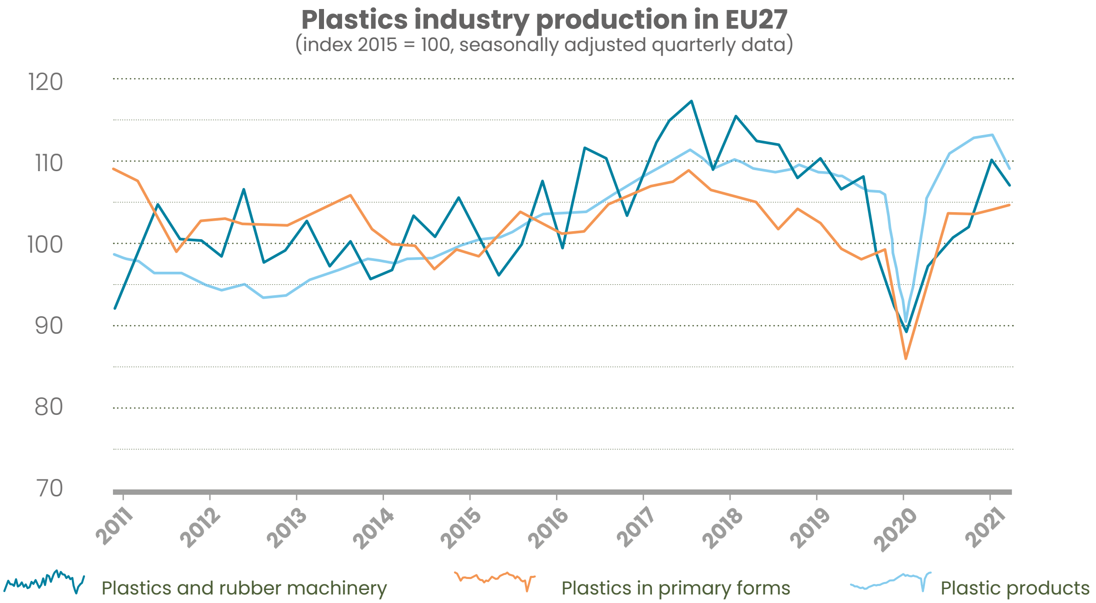

SOURCE: Eurostat.

Evolution of the production of

PLASTICS IN PRIMARY FORMS

European plastics manufacturers benefited from the high demand for plastics in 2021 due to the global economic upswing. However, many companies had to deal with supply chain disruptions, bottlenecks in precursors, and rising energy prices.

Production of plastics in primary forms, EU27

Index 2015 = 100 on a quarterly basis; seasonally and working day adjusted.

Growth rates

2019	-3.6%
2020	-5.2%
2021 forecast	+8.5%

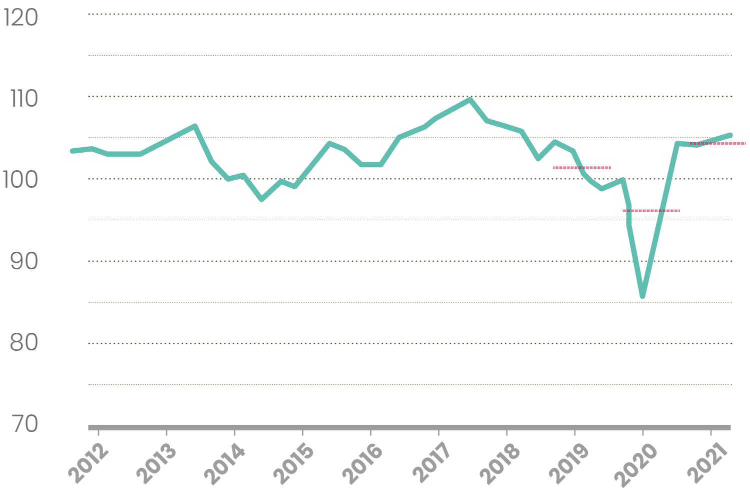

Production primary plastics

Average annual index

SOURCE: Eurostat.

GLOSSARY of terms

**ABS**	Acrylonitrile butadiene styrene resin
**ASA**	Acrylonitrile styrene acrylate resin
**bn**	Billion
**CAGR**	Compound Annual Growth Rate
**CIS**	Commonwealth of Independent States
**Conversio**	Conversio Market & Strategy GmbH
**EU**	European Union
**EU27+3**	EU Member States + Norway, Switzerland and the United Kingdom
**EPRO**	European Association of Plastics Recycling and Recovery Organisations
**Mt**	Million tonnes
**NAFTA**	North American Free Trade Agreement
**Other plastics**	Thermosets, adhesives, coatings and sealants
**Other thermoplastics**	Includes polyacetals (e.g. POM), polyesters excl. fibres (e.g. PBT), ASA, EPDM / EPM and further thermoplastics not shown separately
**PA**	Polyamides. Plastics the Facts demand figures only cover PA6 and PA6.6
**PBT**	Polybutylene terephthalate
**PC**	Polycarbonate
**PE**	Polyethylene
**PE-HD**	Polyethylene, high density
**PE-LD**	Polyethylene, low density
**PE-LLD**	Polyethylene, linear low density
**PE-MD**	Polyethylene, medium density
**PEMRG**	Plastics Europe Market Research Group
**PET**	Polyethylene terephthalate
**Plastics in primary forms**	Virgin plastics
**PMMA**	Polymethyl methacrylate
**POM**	Polyoxymethylene
**PP**	Polypropylene
**PS**	Polystyrene
**PS-E**	Expandable polystyrene
**PTFE**	Polytetrafluoroethylene
**PUR**	Polyurethane
**PVC**	Polyvinyl chloride
**SAN**	Styrene-acrylonitrile copolymer
**Thermosets**	Urea-formaldehyde foam, melamine resin, polyester resins, epoxy resins, etc.

Plastics Europe

Plastics Europe is the pan-European association of plastics manufacturers with offices across Europe. For over 100 years, science and innovation has been the DNA that cuts across our industry. With close to 100 members producing over 90% of all polymers across Europe, we are the catalyst for the industry with a responsibility to openly engage with stakeholders and deliver solutions which are safe, circular and sustainable. We are committed to implementing long-lasting positive change.

EPRO
European Association of Plastics Recycling and Recovery Organisations

EPRO is a pan-European partnership of specialist organisations that are able to develop and deliver efficient solutions for the sustainable management of plastic waste, now and for the future. EPRO members are working to optimise national effectiveness through international co-operation: by studying successful approaches, evaluating different solutions and examining obstacles to progress. By working together EPRO members can achieve synergies that will increase efficient plastics recycling and recovery. Currently 19 organisations in 14 European countries, South Africa and Canada are represented in EPRO.

PLASTICS EUROPE
Enabling a sustainable future

Rue Belliard 40, box 16
1040 Brussels – Belgium
☏ +32 (0)2 792 30 99

connect@plasticseurope.org
www.plasticseurope.org

@PlasticsEurope

PlasticsEurope

epro
European Association Of Plastics Recycling & Recovery Organisations

Konigin Astridlaan 59, bus 5
1780 Wemmel – Belgium
☏ +32 (0)2 456 84 49

info@epro-plasticsrecycling.org
www.epro-plasticsrecycling.org

© 2021 Plastics Europe • All rights reserved.
